# APOBEC3H structure reveals an unusual mechanism of interaction with duplex RNA

**DOI:** 10.1038/s41467-017-01309-6

**Published:** 2017-10-18

**Authors:** Jennifer A. Bohn, Keyur Thummar, Ashley York, Alice Raymond, W. Clay Brown, Paul D. Bieniasz, Theodora Hatziioannou, Janet L. Smith

**Affiliations:** 1 Life Sciences Institute, University of Michigan, Ann Arbor, MI 48109 USA; 2 Department of Biological Chemistry, University of Michigan, Ann Arbor, MI 48109 USA; 3 Laboratory of Retrovirology, The Rockefeller University, New York, NY 10065 USA; 4 Howard Hughes Medical Institute, The Rockefeller University, New York, NY 10065 USA

## Abstract

The APOBEC3 family of cytidine deaminases cause lethal hypermutation of retroviruses via deamination of newly reverse-transcribed viral DNA. Their ability to bind RNA is essential for virion infiltration and antiviral activity, yet the mechanisms of viral RNA recognition are unknown. By screening naturally occurring, polymorphic, non-human primate APOBEC3H variants for biological and crystallization properties, we obtained a 2.24-Å crystal structure of pig-tailed macaque APOBEC3H with bound RNA. Here, we report that APOBEC3H forms a dimer around a short RNA duplex and, despite the bound RNA, has potent cytidine deaminase activity. The structure reveals an unusual RNA-binding mode in which two APOBEC3H molecules at opposite ends of a seven-base-pair duplex interact extensively with both RNA strands, but form no protein–protein contacts. CLIP-seq analysis revealed that APOBEC3H preferentially binds to sequences in the viral genome predicted to contain duplexes, a property that may facilitate both virion incorporation and catalytic activity.

## Abstract (web summary)

The APOBEC3 family cytidine deaminases with antiviral activity are proteins that catalyze the deamination of newly reverse-transcribed viral DNA. Here the authors present the crystal structure of full-length pig-tailed macaque APOBEC3H with bound RNA, which reveals how the APOBEC3H dimer binds around a short RNA duplex.

## Introduction

APOBEC3 (apolipoprotein B mRNA-editing enzyme, catalytic polypeptide-like, family 3; “A3”) antiviral proteins^1^ cause lethal G-to-A hypermutation in retroviruses and retroelements by converting deoxycytosine to deoxyuracil in newly reverse-transcribed single-stranded DNA (ssDNA)^2^. Primate genomes encode seven A3 proteins, of which at least three (A3F, A3G, and A3H) inhibit human immunodeficiency virus (HIV-1) replication, but are antagonized by the viral Vif accessory protein in a species-dependent manner^3–5^. A3 proteins are comprised of one (A3H) or two (A3F and A3G) “Z” domains containing Zn-coordinating His and Cys amino acids that, together with a conserved Glu, form the deaminase catalytic center^2^. Among the three identified Z-domain clades (Z1–Z3), A3F and A3G are comprised of Z1 and Z2 domains, while A3H is unique among primate A3 proteins in possessing a single Z3 domain^2^.

RNA binding is a key feature of A3 proteins that is necessary for their encapsidation into virions and for antiviral activity^6,7^. Although the mechanisms by which A3s recognize RNA are unknown, they are sufficiently non-specific that widely divergent RNA sequences are recognized, yet they exhibit sufficient selectivity to enable virion RNA binding and incorporation in the presence of a vast excess of cellular RNA^8,9^. A3 binding to RNA may also contribute to the deaminase-independent ability to inhibit HIV-1 reverse transcriptase (RT)^10–13^. In addition, A3H and the RNA-binding domains of A3F and A3G drive the formation of higher-order oligomeric complexes^14,15^. RNA binding is thought to inhibit deaminase activity, via an unknown mechanism^13,16,17^, and in the two-domain A3F and A3G proteins, one Z-domain binds RNA and the other catalyzes cytidine deamination^2^. Crystal structures obtained for the isolated catalytic domains of A3F and A3G and for an A3G RNA-binding domain reveal a common domain structure and Zn-binding site^18–22^. Nevertheless, in each case, the A3 protein was extensively truncated or engineered to improve stability or crystallization properties. The structure of an unmodified huA3C has also been obtained^23^, however, this protein lacks antiviral activity. Therefore, thus far, no structure is available for a naturally occurring, full-length APOBEC3 with antiviral activity, and the structural basis for RNA recognition by any APOBEC3 is unknown.

Here, we present the 2.24-Å crystal structure of a full-length, natural polymorphic variant of pig-tailed macaque APOBEC3H (pgtA3H) bound to a short RNA duplex. Furthermore, we show that human A3H haplotype II (huA3H) and several pgtA3H variants retain cytidine deaminase activity in the presence of bound RNA, demonstrating that the RNA does not obstruct access to the A3H active site. CLIP-seq analysis reveals that the regions of the viral genome most frequently bound by A3H are predicted to form short duplex structures. These results illuminate important elements of RNA recognition and potential mechanisms of antiviral activity by a natural, catalytically competent pgtA3H.

## Results

### Naturally occurring polymorphisms in pig-tailed macaque A3H

**Fig. 1:**
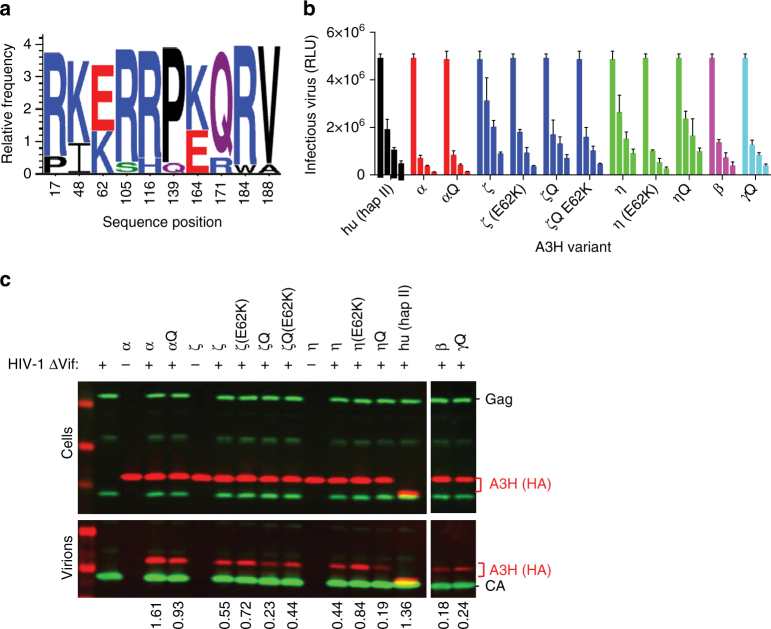
A3H amino acid polymorphism. **a** Logo representation of A3H variants obtained from 14 pigtail macaques. Ten sites of variation were identified and at least 13 allelic variants were identified due to variation at multiple sites in some animals (Supplementary Fig. 1). **b** Antiviral activity of pgtA3H variants compared to huA3H (hap II). HIV-1ΔVif stocks were generated in the presence of increasing amounts of each HA-tagged A3H variant (0, 37, 75, 150 ng expression plasmid) and titrated on indicator cell lines expressing nanoluciferase under the control of the HIV promoter. Luciferase activity was measured and expressed as relative light units (RLUs). Mean and standard deviation of *n* = 2 independent experiments. **c** Virion incorporation of A3H variants. 293T cells stably expressing HA-tagged A3H variants were infected with HIV-1ΔVif. Infected cells and sucrose purified virion particles were analyzed by immunoblot. The specificity of virion incorporation was verified using supernatants from uninfected cells expressing selected A3H variants. Levels of A3H virion incorporation are shown under the gels and expressed as the ratio of the signals (arbitrary units) of A3H bands over viral capsid protein (CA) bands

A3H is an attractive target for biochemical and structural study because the single Z3 domain possesses all three biochemical activities: cytidine deaminase, RNA binding, and Vif binding. Seven A3H haplotypes (hap) have been identified in humans, but only three (hap II, hap V, hap VII) restrict HIV-1^24,25^. Because our initial attempts to crystallize human (hu) A3H hap II were unsuccessful, we explored natural A3H variants encoded by non-human primates, specifically pig-tailed macaques (pgt). Remarkably, analysis of pgtA3H sequences from 14 macaques revealed no less than 13 allelic variants, each of which was expressed as two spliced isoforms that included or excluded a Gln182 codon at the exon 4/5 boundary (26 natural protein variants, Fig. 1a, Supplementary Fig. 1). Analysis of eight naturally occurring pgtA3H protein variants revealed similar expression levels in transfected cells and modest variations in levels of virion encapsidation, and in antiviral activity, compared to huA3H (hap II) (Fig. 1b, c). Specifically, greater antiviral potency was generally associated with pgtA3H variants encoding Lys62 (*α*, *β*, *γ*) as opposed to Glu62 (*ζ*, *η*), and introduction of Lys62 in variants *ζ* and *η* increased their antiviral activity (Fig. 1b). In addition, the presence of Gln182 marginally enhanced antiviral activity in variant *ζ* (Fig. 1b). The levels of antiviral activity generally correlated with the levels of incorporation into particles.

### Cytidine deaminase activity of A3H

**Fig. 2:**
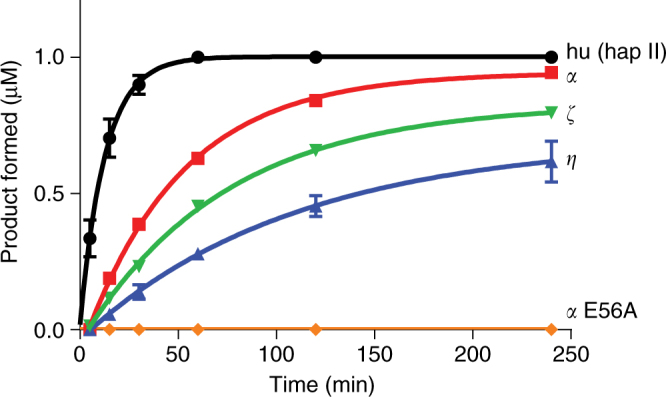
Cytidine deaminase activity of recombinant A3H variants. Single-stranded DNA (1 μM) containing a single reactive cytosine base was combined with 100 nM A3H coupled with 40 nM uracil deglycosylase, and products were detected by denaturing gel electrophoresis. The active site mutant pgtA3Hα E56A^13^ was used as a negative control for A3H deaminase activity; no activity was detected. Mean and standard deviation of *n* = 3 experiments. Initial rates are reported in Table 1

Recombinant full-length, native-sequence huA3H, pgtA3Hα, pgtA3Hζ, and pgtA3Hη expressed in bacteria adopted a mixture of oligomeric states, as detected by gel filtration (Supplementary Fig. 2a), and co-purified with a substantial amount of nucleic acid that was sensitive to RNase but not DNase. Attempts to remove the RNA were not successful, but extensive RNase A treatment resulted in a monodisperse preparation with an apparent molecular weight of 60 kDa, suggesting the presence of a dimeric complex of 25.2 kDa polypeptides (Supplementary Fig. 2b, c). This is consistent with a report of the oligomer state of a recombinant MBP-huA3H fusion protein from an *Escherichia coli* expression system^26^ and recombinant huA3H from an insect-cell expression system^27^. The complex also contained bound RNA with a length of 10–12 nt, determined by denaturing gel electrophoresis (Supplementary Fig. 2d). All purified A3Hs were catalytically active on a 40-nt ssDNA substrate, in which the reactive cytosine is preceded by a thymidine (5′-TC-3′) (Fig. 2, Table 1, Supplementary Fig. 3a, Supplementary Note 1). The differences in the in vitro deaminase activity between the A3H proteins did not precisely correlate with the antiviral activity of each protein (compare Fig. 1b to Fig. 2, Table 1). This could be attributed to: (1) deaminase-independent mechanisms of viral inhibition^13^ that may differ among the A3H variants, and (2) the use of the same 40-nt DNA substrate for the in vitro deaminase assays that may not account for any subtle differences in substrate sequence preference between the A3H variants. For example, the activity of huA3H (hap II) varied by twofold with substrates differing in the +2 position relative to the reactive cytosine^26^. The variation in activity was not due to differences in Zn incorporation among the A3H variants. We confirmed that each A3H variant was purified with a full complement of Zn by comparing activities of samples with and without added Zn. For each variant, the deaminase activity was identical in parallel assays with and without added Zn (Supplementary Fig. 3b).

**Table 1:** Initial rates of A3H cytidine deamination

A3H	Initial rate (per min)
hu (hap II)	1.46 ± 0.01
pgtα	0.15 ± 0.01
pgtζ	0.087 ± 0.006
pgtη	0.055 ± 0.008
pgtα E56A	No activity

The ability of all purified A3H variants with bound RNA to maintain deaminase activity demonstrates that the bound RNA does not obstruct access of DNA to the active site, indicating that it is possible for both DNA and RNA oligonucleotides to bind A3H simultaneously. We further tested whether ribonuclease treatment is critical to deaminase activity, as has been reported for A3H^13^ and A3G^10–12^. The huA3H and pgtA3Hζ were purified in absence of added RNase A. Duplicate samples were then incubated with or without RNase A and assayed. All samples had substantial deaminase activity, with twofold greater activity in the ribonuclease-treated A3H. (Supplementary Fig. 3c, Supplementary Note 1).

### Structure of a pgtA3H-RNA complex

**Fig. 3:**
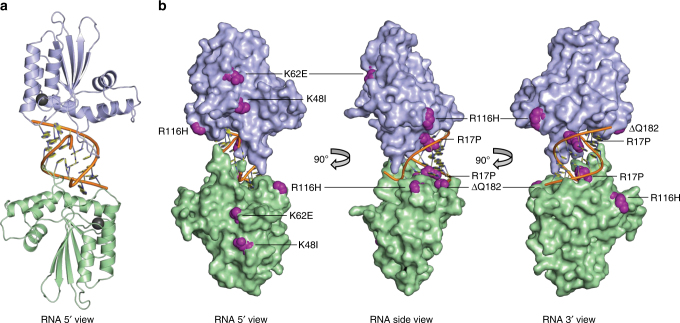
Crystal structure of pgtA3Hζ-RNA complex. **a** Two A3H molecules (blue and green cartoon, Zn as gray sphere) at the ends of a central RNA duplex (orange backbone, yellow bases). **b** Sites of pgtA3H polymorphic variation. The A3H surface (colored as in A) with sites of variation in magenta. The RNA is nearly engulfed by the two A3H molecules, which have no direct contacts with each other

We attempted crystallization with each of the four purified recombinant A3H variants, but only pgtA3Hζ yielded crystals suitable for structure determination. The pgtA3Hζ structure was solved using the anomalous scattering of the native Zn ion at the active site of the enzyme, resulting in a high-quality 2.24-Å structure (Fig. 3, Table 2). The refined model is complete except for two amino acids at the N-terminus and ~30 amino acids at the C-terminus that are present in some non-human primate A3Hs but not in huA3H (Supplementary Fig. 1)^24^. A3H has the familiar A3 fold composed of a five-stranded central β-sheet surrounded by six α-helices, and is highly similar to structures of other A3s in the overall fold and in the active site^18^ (Supplementary Fig. 4a, b). Compared to the catalytic domains of A3F and A3G and the RNA-binding domain of A3G, A3H has significant differences in loop 1, loop 3 (proximal to the active site and variable among the four A3H molecules in the crystal), and in the 15 amino acids preceding the C-terminal helix (Supplementary Fig. 4b, c). Loop 7, which has been implicated in RNA binding^13,14,17,28,29^, is nearly identical in A3H and the RNA-binding domain of A3G (Supplementary Fig. 4b). A3H residues implicated in the recognition by Vif proteins^30,31^ are clustered on A3H exposed surfaces, specifically at helices α3 and α4 (Supplementary Fig. 5).

**Table 2:** Data collection and refinement statistics

	Native^a^	Zn anomalous^b^
*Data collection*		
Space group	*P*22_1_2_1_	*P*22_1_2_1_
*Cell dimensions*		
*a*, *b*, *c* (Å)	88.0, 89.3, 134.4	90.4, 91.7, 139.3
*α*, *β*, *γ* (°)	90, 90, 90	90, 90, 90
Wavelength (Å)	1.033	1.283
Resolution (Å)	44.6–2.24 (2.32–2.24)	45.8–2.59 (2.68–2.59)^c^
*R*_merge_	0.128 (2.58)	0.086 (1.08)
*R*_PIM_	0.03 (0.50)	0.04 (0.45)
*R*_meas_	0.13 (2.63)	0.09 (1.16)
*I*/*σI*	15.8 (1.09)	15.7 (1.75)
Completeness (%)	1.00 (0.99)	1.00 (0.99)
Redundancy	27.1 (26.4)	7.1 (6.7)
CC_½_	1 (0.48)	1 (0.64)
*Refinement*		
No. of reflections	51,313	
*R*_work_/*R*_free_	0.181/0.222	
*No. of atoms*	7204	
Protein	6120	
RNA	830	
Zn	4	
Water	250	
*Average B-factor (Å* ^*2*^ *)*	84.4	
Protein	85.3	
RNA	79.5	
Zn	78.2	
Water	78.3	
*R.m.s deviations*		
Bond lengths (Å)	0.002	
Bond angles (°)	0.53	

^a^Data merged from two crystals

^b^Anomalous pairs were kept separate for calculating statistics

^c^Values in parentheses pertain to the outermost shell of data

### Binding of A3H to the RNA duplex

Surprisingly, A3H was bound not to ssRNA, but to an RNA duplex. Two A3H molecules form a complex with one RNA duplex (Fig. 3a, b). The crystals contain two such A3H–RNA complexes, which are identical within experimental error excepting the variability in loop 3, which does not contact RNA. The model-unbiased method of structure determination resulted in excellent electron density for the RNA duplex, including the base, ribose, and phosphate for nine nucleotides in each strand (Supplementary Fig. 6a). Refinement after removal of the ribose-specific 2′-hydroxyl atoms from the model resulted in difference electron density for the 2′-hydroxyls, confirming that both strands of the duplex are RNA (Supplementary Fig. 6b). The A3H–RNA complex is maintained solely through protein–RNA contacts; there are no direct contacts between A3H protein molecules. Rather, each A3H molecule contacts both strands of the RNA duplex, and together the two A3Hs bury >40% of the RNA duplex surface area with a total of 1090 Å^2^ buried at the RNA interface of each A3H molecule (2180 Å^2^ total in the complex) (Fig. 3).

**Fig. 4:**
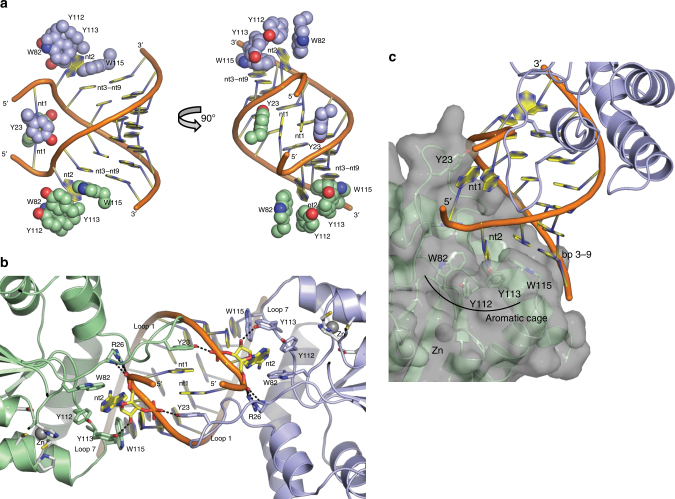
Unusual A3H–RNA interaction. **a** Aromatic amino acids (spheres with C colored as in Fig. 2a) that contact RNA bases (cartoon). The nt1 bases stack with each other and with Tyr23, the nt2 bases are bound in an “aromatic cage”, and the nt3–nt9 base pairs stack with Trp115. **b** Loop 1 protrusion into the RNA narrow major groove. Key amino acids and nucleotides are shown in stick form (green or light blue C, red O, blue N, orange P) with hydrogen bonds (dashed lines) from Tyr23, Arg26, and Tyr113 to the RNA. Side chains in the active sites are shown in stick form with white C atoms and Zn ions as gray spheres and correspond to the zinc-dependent deaminase signature motif (C/H)xE(x)_*n*_PCxxC (His54, Glu56, Pro84, Cys85, Cys88). **c** Surface view of one A3H molecule showing the pocket formed by the “aromatic cage” for the unpaired nt2 base and the nearby active site entrance

The A-form duplex in the A3H complex is typical for RNA (Supplementary Fig. 7a) and for RNA/DNA heteroduplexes (Supplementary Fig. 7b). Of the nine nucleotides in each strand (nt1–9), seven are base paired (nt3–9) and the two 5′ nucleotides (nt1 and nt2) are unpaired. The 3′-end of the RNA terminates in the nt3–nt9 base pair, which is capped by stacking with Trp115 in A3H loop 7 (Fig. 4a) within the _110_RLYYHW_115_ putative RNA-binding motif^13,14,17,28,29^. A sharp kink in the RNA between nt1 and nt2 brings the 5′ nucleotides close to one another and results in stacking of the nt1 bases, which are sandwiched between the phenolic rings of Tyr23 in the two A3H molecules (Fig. 4a). The RNA kink is stabilized by A3H loop 1 (residues 13–30), which inserts into the narrow major groove of the A-form RNA duplex and includes a unique four-residue insertion (_22_PYYP_25_) (Fig. 4b). At the tip of loop 1 in each A3H molecule, *cis*-Pro22 positions Tyr23 to stack with the nt1 base of one RNA strand, and Arg26 stabilizes the sharp RNA kink through three hydrogen bonds to the phosphate at the nt1–nt2 bend (Fig. 4b). The only hydrogen bond of A3H to RNA not involving a phosphate is between the Tyr113 hydroxyl and the nt2 ribose 2′-hydroxyl (Fig. 4b). The unpaired nt2 base protrudes into an A3H “aromatic cage” composed of amino acids from loop 7 (Tyr112, Tyr113, and Trp115) and loop 3 (Trp82) (Fig. 4a, c). These tryptophan and tyrosine side chains form a network of favorable edge-to-face interactions with the nt2 base, akin to typical interactions of aromatic side chains in protein interiors. In addition to many of these specific interactions, several arginine and lysine side chains hydrogen bond with RNA phosphates and impart a strongly basic character to the A3H surface that contacts RNA (Supplementary Fig. 8).

In total, each A3H molecule forms nine hydrogen bonds with the phosphate backbone (six to one strand and three to the other), one hydrogen bond to the nt2 2′-hydroxyl, and aromatic interactions with bases in both RNA strands (nt1, nt2, nt3–9 base pair) (Fig. 4). There are no base-specific contacts, consistent with the expected lack of sequence specificity in RNA binding^8,9^. Accordingly, the co-purified RNA lacks a distinct sequence. For each of the seven base pairs, electron density is continuous across the paired bases (Supplementary Fig. 6), that is, either base could be a purine or pyrimidine. In summary, A3H is selective for RNA through a strong conformational preference for an A-form duplex with seven base pairs, but it lacks sequence specificity.

### Binding of A3H to RNA duplexes in the viral genome

**Fig. 5:**
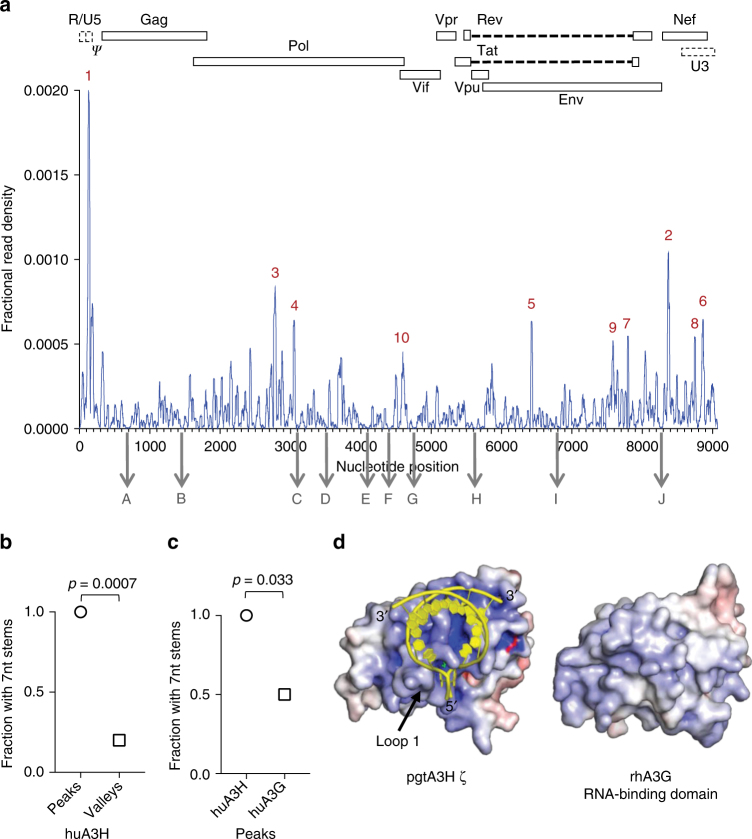
Analysis of A3H binding to the HIV-1 genome. **a** Frequency distribution of nucleotide occurrence (read density) relative to the sum of read densities in reads mapped to the HIV-1 NL4-3 genome in a huA3H CLIP-Seq experiment using HIV-1 particles^8^. The 10 most frequently (designated 1–10 for highest to lowest fractional read density) and 10 least frequently bound sites (designated A–J for relative position in the viral genome) are indicated. A schematic diagram of the HIV-1 genome is shown above. **b** Frequency with which ≥7-nt duplexes are predicted to occur within 101-nt RNA elements containing the 10 sites with high-frequency binding (peaks 1–10), and 101-nt elements with low-frequency binding (valleys A–J). **c** Frequency with which ≥7-nt duplexes are predicted to occur within 101-nt RNA elements containing the 10 sites most frequently bound by A3H and A3G. **d** Comparison of surface charge distribution for A3H and the A3G RNA-binding domain. RNA (yellow cartoon) binds to the most positively charged surface of A3H (electrostatic surface potential colored from red to blue, −10 to +10 kT/e). The analogous surface of the A3G RNA-binding domain (PDB 5K81^22^) is relatively hydrophobic

The A3H:RNA complex structure represents a binding mode that should be relevant to the A3H–RNA interaction in virions, leading to the prediction that A3H may bind preferentially to 7 nt RNA duplexes in the viral genome during encapsidation. Because amino acids that contact RNA are conserved in huA3H and pgtA3H, we analyzed CLIP-Seq data obtained using virion-encapsidated huA3H^8^. The ten most frequently bound sites in the viral genome all contained one or two predicted RNA duplexes of seven or more nucleotides (Fig. 5a, Supplementary Fig. 9), while only 2 of 10 control sites with low-frequency A3H binding contained predicted 7 nt duplexes (*p* = 0.0007, Fig. 5b). The HIV-1 5′ leader, particularly, the poly-A stem loop and sequences surrounding the primer-binding site were prominent sites of A3H binding (Fig. 5a). This portion of the genome contains extensive RNA duplexes, including the genome-transfer RNA primer complex^32^. A similar CLIP analysis of huA3H and the three pgtA3H protein variants collected from infected cells rather than virions (Supplementary Fig. 10a) also indicated a statistically significant preference for areas in the viral genome that contain potential duplexes of ≥7 nt (Supplementary Fig. 10b). Comparison of the nucleotide compositions of favored binding sites (peaks) vs. disfavored sites (valleys) indicated relative enrichment of G-nucleotides in peaks (even though the HIV-1 genome is A-rich) and A-nucleotides in valleys (Supplementary Fig. 11). This characteristic is consistent with the notion that A3H favors binding to transiently formed duplexes, promoted in G-rich regions by the ability of G to pair with both U and C, in otherwise predominantly ssRNA molecules.

## Discussion

We found an unusually high degree of polymorphism in A3H proteins in a non-human species and have demonstrated that all variants exhibit potent antiviral activity. Importantly, this polymorphism allowed the identification of a naturally occurring A3H protein that is amenable to crystallization. We have determined the structure of a full-length, unaltered, APOBEC3 protein with antiviral activity. Furthermore, A3H unexpectedly cocrystallized with duplex RNA, thus the structure, in combination with CLIP-seq data, illuminates how A3H could be encapsidated into virions and how it inhibits viral replication independent of cytidine deamination^13^.

An understanding of how APOBEC3 proteins are specifically incorporated into virions has been elusive, given their apparent lack of specificity in recognition of RNA sequences, and the abundance of cellular RNAs in the cytoplasm, only a tiny fraction of which are packaged into virions. Our data favor a model in which the A3H protein preferentially binds to short RNA duplexes with little or no primary sequence discrimination during encapsidation. While other binding modes may be possible, favoring short duplex RNA in this manner might provide selectivity for viral RNA molecules that are in the act of compacting during virion packaging. Selective binding to only a sample of all possible RNA sequences, that is, those that are capable of duplex formation, should also reduce the effect of “distracting” cellular RNAs. It is also noteworthy that the observed prominent binding in the structured 5′ leader would position A3H optimally for the inhibition of reverse transcription^13^. As the 5′ leader is the most frequently bound site within the HIV-1 genome in virions, this could account for the deaminase-independent antiviral activity of A3H^13^. Intriguingly, the A3H–RNA-binding mode is equally relevant to an RNA-DNA heteroduplex (Supplementary Fig. 7b) such as the initial product of reverse transcription in which the RNA strand is cleaved at ~6-nt intervals by RNase H^33^. The active site for cytidine deamination is near to the RNA 5′ end (Fig. 4b, c), suggesting that A3H binding to the RNA-DNA heteroduplex product of reverse transcription may be an evolved property that facilitates delivery of DNA to the active site.

Our results demonstrate that, while ribonuclease treatment increased the rate of deamination by twofold (Supplementary Fig. 3c), it is not essential to the deaminase activity of purified A3H. This is in contrast to Mitra et al.^13^, who found that RNase A treatment was required for activity of huA3H in mammalian cell extracts. Thus, it is likely that purification—with or without ribonuclease treatment—removed interfering RNA molecules and that the RNA fragments remaining bound to A3H do not significantly inhibit activity.

APOBEC3 family members are composed of Z domains of similar overall structure (Supplementary Fig. 4a), including a highly conserved characteristic Zn-binding site. Nevertheless, in double domain APOBEC3 proteins, such as A3G and A3F, each domain has adopted a distinct function in which the N-terminal domain (NTD) binds viral RNA and the C-terminal domain (CTD) exhibits enzymatic activity. Furthermore, while the NTD in A3G is targeted by Vif, the CTD is targeted in A3F^18,34^. In contrast, in A3H, a single domain exhibits all these functions. Multiple A3H residues that cluster on the surface of helices α3 and α4 have been implicated in Vif binding (Supplementary Fig. 5). A3H polymorphism in humans appears to have driven HIV-1 Vif evolution^35,36^. Interestingly, HIV-1 Vif does not recognize macaque A3H, while HIV-2 and SIVmac Vif do^3–5^. Residues that differ between human and macaque A3H (Supplementary Fig. 1) are scattered across the protein surface. It will be interesting to determine which of these residues governs differential Vif recognition.

Despite the similarities in overall structure with other members of the APOBEC3 family and the strongly basic character of the RNA-binding Z domains (A3H, A3F NTD, A3G NTD), the unusual mode of RNA binding by A3H appears to be distinct. Indeed, the 10 most frequent A3G-binding sites in HIV-1 virion RNA were significantly less likely to contain predicted, overlapping ≥7 nt duplexes than were the preferred A3H-binding sites (*p* = 0.033, Fig. 5c). In addition, whereas all A3H variants bind strongly to the 5′ leader of the viral genome (Fig. 5a, Supplementary Figs. 9, 10), A3G does not, and the overall distribution of peaks and valleys in virions differs between the two proteins^8^. Accordingly, RNA-binding features in A3H (Fig. 4), particularly a loop 1 insertion that contacts the RNA narrow major groove and the overall surface charge distribution, are clearly different from the A3G NTD (Fig. 5d, Supplementary Fig. 12). Identical basic surfaces of two A3H molecules contact a short RNA duplex to form an A3H–RNA complex, whereas the analogous A3G NTD surface is hydrophobic and forms a protein dimer^22^, strongly suggesting heterogeneity in the ways in which different A3 proteins recognize RNA.

Overall, the A3H:RNA complex structure presented herein provides an unexpected view of an unusual RNA-binding mode for A3H and illuminates potential mechanisms of antiviral activity.

## Methods

### Identification of APOBEC3H variants in pig-tailed macaques

Total RNA and genomic DNA were extracted from 14 activated, IFNα-treated pig-tailed macaque peripheral blood mononuclear cell samples using TRIZOL (GibcoBRL). Thereafter, complementary DNA (cDNA) was synthesized using the SuperScript III RT kit (Invitrogen) using gene-specific primers (Supplementary Table 1) based on the rhesus macaque A3H sequence (NM_001042372). Two independent PCR reactions were performed on each cDNA sample, introduced into pCR-Blunt (Invitrogen) and a total of 12–26 clones were analyzed by sequencing. Selected clones were subcloned into a modified LHCX (Clontech) vector, that expresses hygromycin, such that the A3H stop codon was removed and three copies of an HA tag were fused to the C-terminus of the protein (A3H-3xHA)^8^. Next, 800 ng of each A3H-3xHA expressing plasmid or the LHCX control plasmid were co-transfected in 293T cells (ATCC) (8 × 10^5^ cells per well—six-well) using 12 μL polyethylenimine (PEI) (Polysciences, Inc.) together with 800 ng of a murine leukemia virus (MLV) GagPol expression plasmid and 100 ng of a vesicular stomatitis virus glycoprotein (VSV.G) expression plasmid to produce MLV-based retroviral stocks, which were subsequently used to transduce 293T cells. Cells stably expressing each pig-tailed A3H (pgtA3H) variant were selected and maintained in medium containing hygromycin (10 ng/mL)^8^.

### Virion incorporation assay

293T cells stably expressing huA3H or pgtA3H variants (7 × 10^5^ cells per well—six-well) were infected with VSV.G pseudotyped HIV-1_NL4.3ΔVif_ at a multiplicity-of-infection (MOI) of 2. At 16 h post infection, supernatants were collected, clarified by centrifugation at 748×*g*, filtered through a 0.22 μm filter, and virions were purified via ultracentrifugation through a 25% sucrose cushion. Supernatants from uninfected A3H-expressing cells were used as control for specificity of A3H incorporation into viral particles. Purified virions and cell lysates were analyzed by immunoblot using a rabbit anti-HA at a 1:1000 dilution (600-401-384 Rockland) and a mouse anti-HIV-1 p24 capsid at a 1:100 dilution (183-H12-5C, NIH AIDS Reagent Program) antibodies, followed by an anti-rabbit IgG conjugated to IR Dye680 and an anti-mouse IgG conjugated to IR Dye800 (LiCOR).

### Antiviral activity assay for A3H variants

293T cells (5 × 10^4^ cell per well—96-well) were transfected using 2 μL PEI (Polysciences, Inc.) with 50 ng of an HIV-1_NL4.3ΔVif_ proviral plasmid, 12 ng of a VSV.G expression plasmid, and 0, 37.5, 75, or 150 ng of each A3H-3xHA expression plasmid using the LHCX plasmid as filler to maintain a constant total amount of DNA per transfection. At 48 h post transfection, supernatant was collected, frozen at −70 °C and then thawed and titrated on Helios indicator cells that constitutively express CD4 and CCR5 and also express a nanoluciferase gene under the control of the HIV-2 LTR. At 48 h post inoculation, infectivity was quantified by measuring nanoluciferase expression using the Nano-Glo Luciferase assay (Promega) and is expressed as Relative Light Units (RLU).

### Expression of A3H protein variants

Recombinant huA3H, three pgtA3H variants (*α*, *η*, *ζ*), and catalytically inactive pgtA3Hα E56A^13^ were produced in *E. coli* using codon-optimized synthetic DNAs (Supplementary Table 1). Ligation-independent cloning (LIC) was used with the expression vectors pMCSG7^37^ (N-terminal His_6_ tag and tobacco etch virus (TEV) protease cleavage site), including all pgtA3H variants, or pMocr^38^ (N-terminal His_6_ tag, Mocr, and TEV cleavage site) for huA3H. All sequences were confirmed by Sanger sequencing. Plasmids phA3H001 and ppgtA3H001-003 were transformed into *E. coli* strain BL21(DE3) carrying the pGro7 plasmid for co-expression of chaperones GroES-GroEL (Takada)^39^. Transformed cells were grown in 4% glycerol terrific broth media at 37 °C until the OD_600_ reached 0.6–0.8. Chaperone expression was induced with l-arabinose (1 g/L) at 37 °C for 1 h, then cultures were cooled to 20 °C, induced with 200 µM IPTG, and grown overnight. Cells were collected via centrifugation and stored at −20 °C.

### Mutagenesis

The catalytically inactive variant of pgtA3Hα was generated by PCR and confirmed by sanger sequencing. Primers were designed with the Quikchange primer design tool website (Agilent) and are included in Supplementary Table 1.

### Protein purification

Bacterial cell pellets were resuspended in buffer A (50 mM Tris pH 8.5, 300 mM NaCl, 10% glycerol, 5 mM β-mercaptoethanol) with 2 mM MgCl_2_, 0.5 mg/mL DNaseI, 0.5 mg/mL RNase A, and 0.1 mg/mL lysozyme (lysis conditions). Under these conditions, resuspended cells from a 1-L culture were incubated on ice for at least 1 h, followed by sonication. Supernatants of centrifuged cell lysates were batch-bound to 5 mL Ni-NTA resin (Qiagen) overnight at 4 °C. The resin was washed with 5 column volumes (CVs) of buffer A followed by a 5-CV wash using buffer A with 1 M NaCl and 20 mM imidazole. Protein was eluted with 6-CV buffer A with 200 mM imidazole. Purification tags were removed by overnight incubation with TEV protease (+2 mM dithiothreitol (DTT)) in dialysis against buffer A. Tag-free protein was separated from uncleaved protein and TEV protease with a 5 mL His Trap column (GE Healthcare), and the flow-through was concentrated and incubated for 4 h with 1 mg/mL RNase A at 25 °C. Attempts to remove nucleic acid from the protein, including PEI precipitation, were unsuccessful, however with extensive RNase A treatment, a monodisperse chromatogram was achieved. Concentrated protein was loaded onto a Superdex 16/60 S200 gel filtration column and eluted in 50 mM Tris pH 8, 150 mM NaCl, 5% glycerol, 1 mM Tris(2-carboxyethyl)phosphine. All APOBEC3H proteins eluted as monodisperse complexes with an apparent molecular weight (MW) of 60 kDa (calculated A3H MW = 25.2 kDa). Peak fractions were pooled, concentrated to 10 mg/mL, and flash-frozen in liquid N_2_. All purified proteins, despite removal efforts, contained nucleic acid as shown by the absorbance ratio (*A*_260 nm_/*A*_280 nm_) of 1.5. Tag-free protein was used for all biochemical and crystallographic experiments. No RNase A was used in the purification of proteins for the assay data shown in Supplementary Fig. 3c.

### Nucleic acid extraction and denaturing gel electrophoresis

Nucleic acid was extracted from each A3H preparation after the gel filtration step using a phenol:chloroform extraction followed by ethanol precipitation (95% ethanol for 2 h at −20 °C)^40,41^. Extracted nucleic acids from each A3H preparation were separated on a 15% urea polyacrylamide denaturing gel followed by overnight staining with Stains All (Sigma) to determine the identity of the bound nucleic acid (purple RNA, blue DNA) and its approximate size (by comparison to defined oligonucleotides of length 30, 20, 17, and 10 nt).

### Cytidine deaminase assay

Cytidine deaminase activity was evaluated in a coupled assay with uracil-DNA glycosylase (UDG)^26,42^. Recombinant UDG^43^ was produced in *E. coli* from pUDG001 generated by LIC of a synthetic DNA (Supplementary Table 1) into pMCSG7, and purified by Ni-affinity chromatography. The substrate was a 40-nt ssDNA oligomer containing one deoxycytosine and a 5′ fluorescent tag (6-carboxyfluorescein, 6-FAM): 5′-(6-FAM) AATGAAAGATATAAGACTCAAATTGAAATAGTTAAGATTA-3′. Reaction mixtures (total volume 40 µL) contained 1 µM DNA substrate, 40 nM UDG, 50 mM Tris pH 7.5, 40 mM NaCl, 5 mM MgCl_2_, 1 mM DTT, and 4 nM, 20 nM or 100 nM A3H. Reactions at 37 °C were initiated by addition of A3H. At each time point, the reaction was quenched with two volumes of 0.2 M NaOH followed by heating 15 min at 70 °C to hydrolyze DNA at the deglycosylated nucleotide. After addition of three volumes of formamide loading solution, the samples were heated 10 min at 95 °C, and the products were resolved by 15% urea denaturing polyacrylamide gel electrophoresis. Gels were scanned using a Typhoon fluorescence imager, and bands corresponding to cleaved and uncleaved DNA were quantified using the ImageQuant software (GE Healthcare Life Sciences). To confirm that Zn was not limiting in the deaminase assay, parallel experiments were performed as described above with or without addition of 2 and 10 µM ZnCl_2_ to the assay buffer (2× and 10× molar excess of A3H). Activities were identical within experimental error (Supplementary Fig. 3b).

### Crystallography

Crystals of the full-length pgtA3Hζ (10 mg/mL) were grown at 20 °C by sitting-drop vapor diffusion from a 2:1 mixture of protein stock and reservoir solution (28% polyethylene glycol average MW 4000, 0.1 M MgCl_2_, 0.1 M Tris pH 8.5). Crystals grew in 1–2 days, were collected directly from the growth solution, and were flash-cooled in liquid N_2_. Data were collected at the Advanced Photon Source (APS, Argonne National Laboratory) on GM/CA beamline 23ID-B. Anomalous pair data to 2.6 Å were recorded at the X-ray energy of peak absorption (9.667 keV) above the Zn K-edge (9.6586 keV) in 15° wedges with inverse-beam geometry. Additional data to 2.24 Å, recorded from two crystals, were collected at an X-ray energy of 12 keV. Data were indexed, integrated, and scaled using XDS^44^. Initial crystal screening suggested the possibility of a tetragonal space group (*a* ≈ *b*), and we retained these axial assignments throughout the structure determination, which resulted in a nonstandard primitive orthorhombic space group (*P*22_1_2_1_). Anomalous differences to 3.5 Å were used with AutoSol^45^ in the Phenix suite^46^ to determine the Zn substructure, and for initial single-wavelength anomalous phasing. Phases were refined and extended to 2.6 Å by exploiting the fourfold noncrystallographic symmetry using DM^47^. A 78% complete initial model of the protein was built into the 2.6-Å phase-refined map using AutoBuild, and the remainder of the model, including an RNA duplex, was built manually in coot^48^. Although the RNA density was consistent with a mixture for the base paired nucleotides (nt3–9) and for nt2, for simplicity, the RNA strands were modeled with unique sequences (5′-A-A-C-C-C-G-G-G-G-3′ and 5′-A-A-C-C-C-C-G-G-G-3′). The nt1 nucleotide had clear purine density and was modeled as adenine. For two of the RNA strands, weak density extended beyond nt9, and the ribose for an nt10 was included in the model. The model was refined against the 2.24-Å data using phenix.refine^49^. Structure validation was done with MolProbity^50^. In the final model, 98% of amino acids are in favored regions of the Ramachandran plot, 2% in additionally allowed regions, and none are outliers. Structure images were prepared with PyMOL^51^, electrostatic surface potentials calculated with APBS^52,53^, and buried surface areas calculated with PISA^54–56^.

### CLIP-seq analysis

CLIP-seq analyses^8,57^ were conducted using 293T cells stably expressing 3 × HA-tagged pgtA3H proteins. Cells were infected with VSV.G pseudotyped HIV-1_NL4-3 ΔVif_ at an MOI of 2 and fed with 4-thiouridine (4SU; Sigma) 14 h prior to collection. Thereafter, cells and virions were irradiated, lysed in NP-40 lysis buffer, and the soluble fraction was separated by centrifugation. Lysates were subsequently treated with RNase A (Fermentas) and DNaseI (Roche) and then transferred to ice. Protein G-conjugated magnetic Dynabeads coated with a mouse monoclonal anti-HA antibody were added to lysates. After binding, the beads were washed thoroughly^8,57^. Dephosphorylation was done using calf-intestinal phosphatase (NEB), and the beads were then washed and resuspended in 1 bead volume of polynucleotide kinase (PNK) buffer, [γ-^32^P] ATP and T4 PNK (NEB). Cold ATP was added and the phosphorylation reaction continued. The beads were then washed and resuspended in NuPAGE SDS-PAGE loading buffer to elute protein–RNA adducts. Protein–RNA adducts were separated by SDS-PAGE, transferred to nitrocellulose and detected by autoradiography. RNA was isolated by proteinase K treatment and phenol:chloroform extraction^8,57^. Sequential 3′ and 5′ adapter ligations were then performed on the isolated RNA resulting in RNA of unknown sequence that was flanked by known sequence that contained primer-binding sites for subsequent reverse transcription and PCR-amplification of the cDNA library. Sequencing of the cDNA library was performed on an Illumina HiSeq 2000 platform. The analysis pipeline used the FASTX toolkit (http://hannonlab.cshl.edu/fastx_toolkit/) to process the raw reads prior to mapping them to the HIV-1 viral genome^8,57^. Preferred A3H-binding sites in the viral genome (Peaks) were identified as the positions of the HIV-1 genome with the highest read densities. The 10–12 highest peaks were identified for each A3H protein. Thereafter, 50 nucleotides on either side of the nucleotide with the highest read density value were selected for further analysis. Potential RNA duplexes were detected using M-fold^58^ with default parameters. An equal number of regions with low frequencies of A3H binding (Valleys) were identified as 101-nucleotide stretches in the HIV-1 genome with low read density (<200) and subjected to the same analysis as the peaks. To determine the nucleotide composition of the tips of the peaks, the nucleotides with the highest read density within a peak were selected and the base composition determined. For comparison, we identified short valleys as stretches of nucleotides with identical length to the peak tips in a proximal region of the HIV-1 genome. In each CLIP-seq experiment, approximately two to three peaks had wide tips that rendered the identification of short valleys in the vicinity of those peaks impossible, and therefore distal regions of the genome were used.

### Data availability

Atomic coordinates and structure factors have been deposited in the Protein Data Bank (PDB) database under the accession code 5W3V. The pgtA3H variant sequences identified in these studies have been deposited to GenBank (accession numbers MF509624 through MF509631). Other data are available from the corresponding authors upon reasonable request.

## Electronic supplementary material

Supplementary Information
