# Supplementary material for: APOBEC3H structure reveals an unusual mechanism of interaction with duplex RNA
Source: Nat Commun. 2017 Oct 18;8:1021. doi: 10.1038/s41467-017-01309-6 (PMC5647330; doi:10.1038/s41467-017-01309-6)
Supplement: Supplementary file 1 — Supplementary Information [file 41467_2017_1309_MOESM1_ESM.pdf]

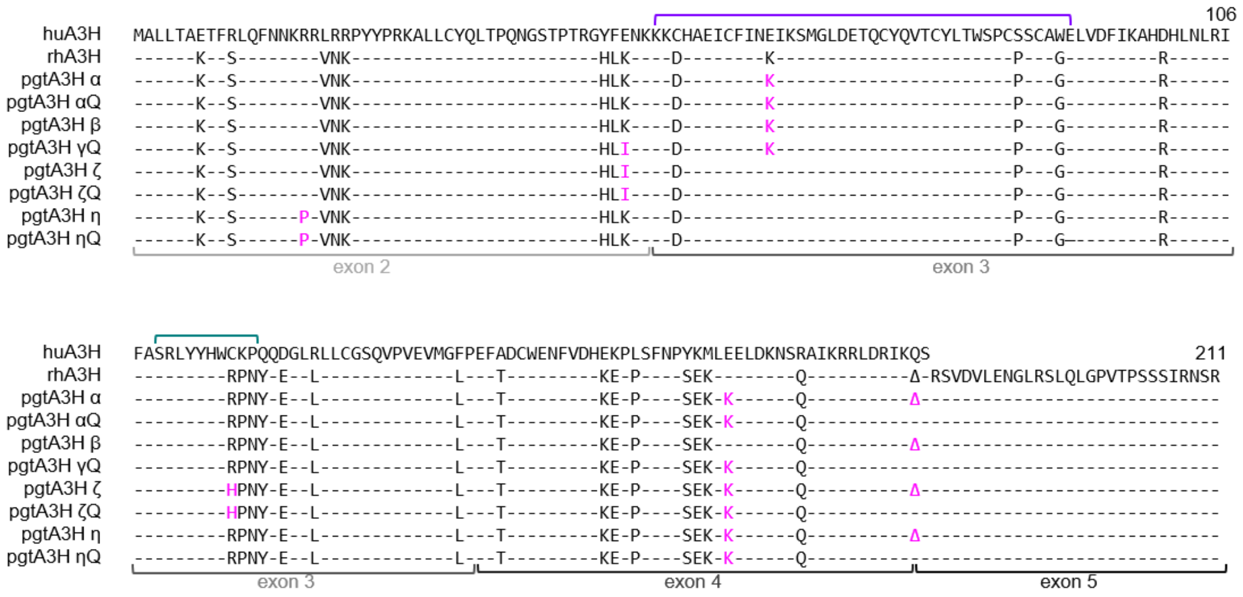

Supplementary Figure 1: Amino acid sequence alignment of pgtA3H and huA3H variants used in these studies and the rhesus A3H variant (rhA3H, NM_001042372).

Residues that differ among the pgtA3H variants are marked in magenta. Protein features are marked above the sequences: the active site region with a purple bar and the site reported to play a role in RNA recognition (loop 7) in teal. Exon positions are marked beneath the sequences.

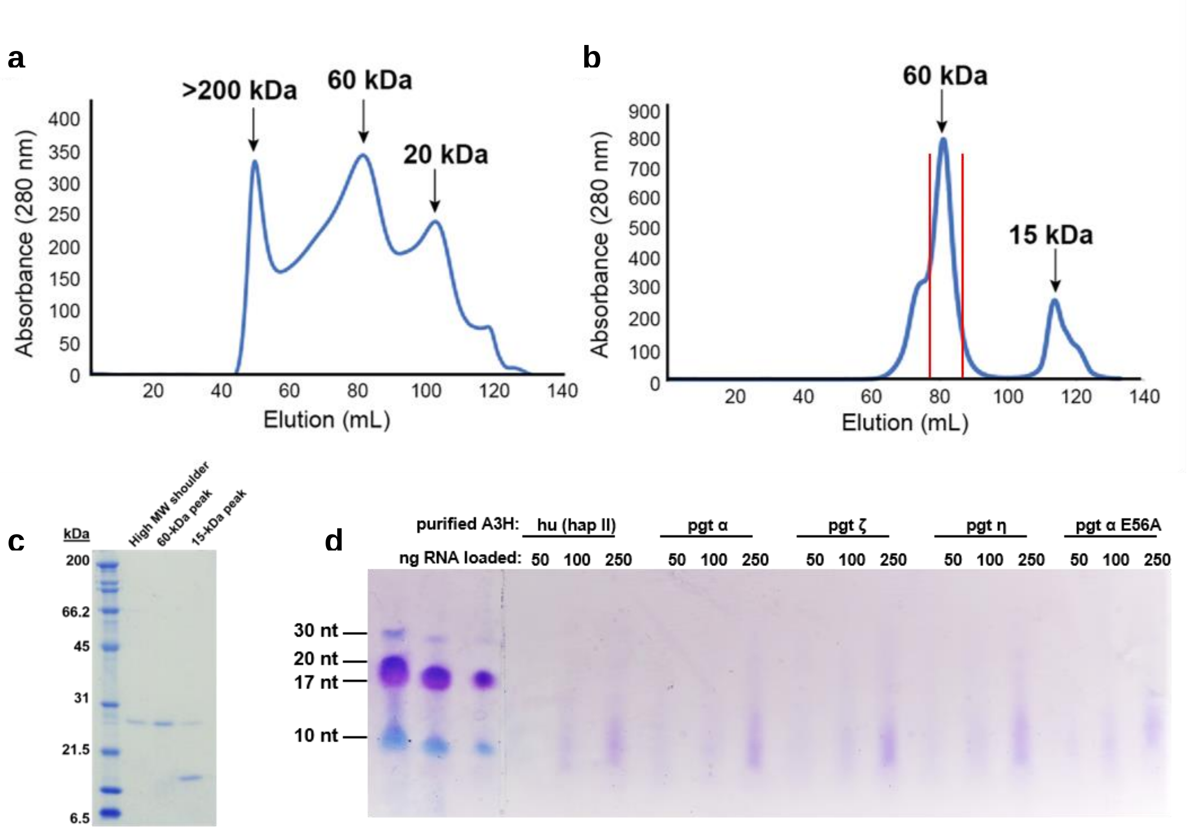

Supplementary Figure 2: Purification of recombinant A3H-RNA.
(a) S200 gel filtration profile of pgtA3Hζ prior to treatment with RNase A, indicating a mixture of oligomeric states. All fractions contained A3H and RNA. (**b)** S200 gel filtration profile of pgtA3Hζ after RNase A digestion, indicating a monodisperse species of apparent molecular weight 60 kDa. Red lines indicate the fractions pooled for biochemical and crystallographic experiments. The fraction that eluted at ~115 mL is predominantly RNase A. **(c)** Coomassie-stained gel of size exclusion fractions (high MW shoulder, 60-kDa peak, and 15-kDa peak in **b**) with MW standards. **(d)** Denaturing gel of RNA extracted from the A3H-RNA peak that eluted at 80 mL (fractions between red lines in **b**). The extracted nucleic acid is RNA, indicated by the purple stain, of heterogeneous length of ~10-12 nt. The monodisperse A3H (fractions between red lines in **b**) with bound RNA (**d)** was used for cytidine deaminase assays and for crystallization.

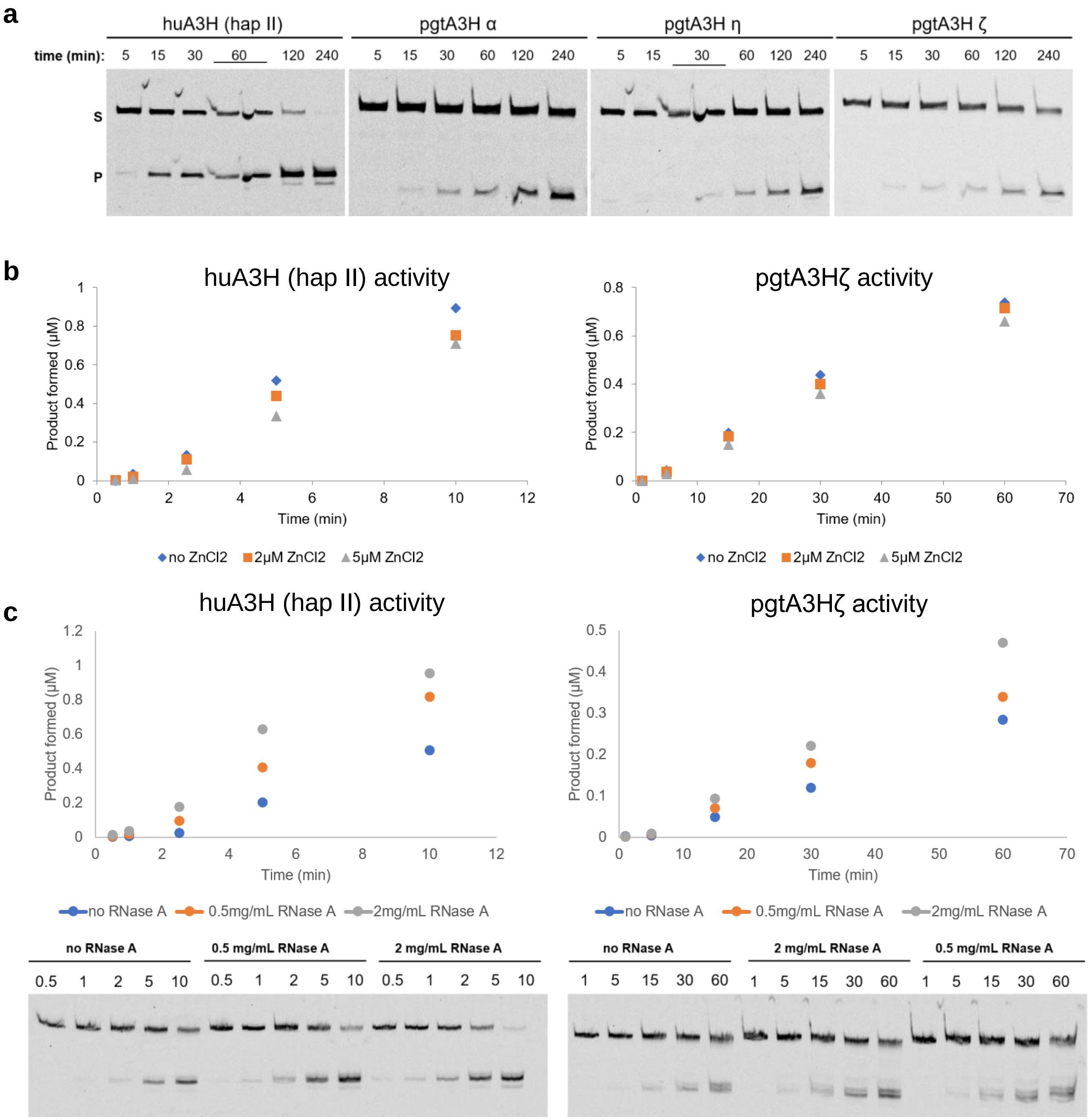

Supplementary Figure 3: Analysis of deaminase activity of A3H variants.
(a) Representative images of deaminase gels for each A3H variant to accompany Fig. 2; S = substrate, P = product. **(b)** Analysis of huA3H (hap II) and pgtA3Hζ deaminase activity with $ZnCl_2$ supplementation at 2x and 10x molar excess of A3H in the reaction mixture. **(c)** Analysis of huA3H (hap II) and pgtA3Hζ deaminase activity with and without RNase A treatment. A3H samples were incubated 1 hr at room temperature without or with RNase A (0.5 mg/mL or 2 mg/mL) before assaying deaminase activity. Both huA3H and pgtA3H proteins are active with and without RNase A treatment. Deaminase gels for each protein are shown below the corresponding time-course plots in the upper panels. Uncropped gel images are shown in Supplementary Note 1.

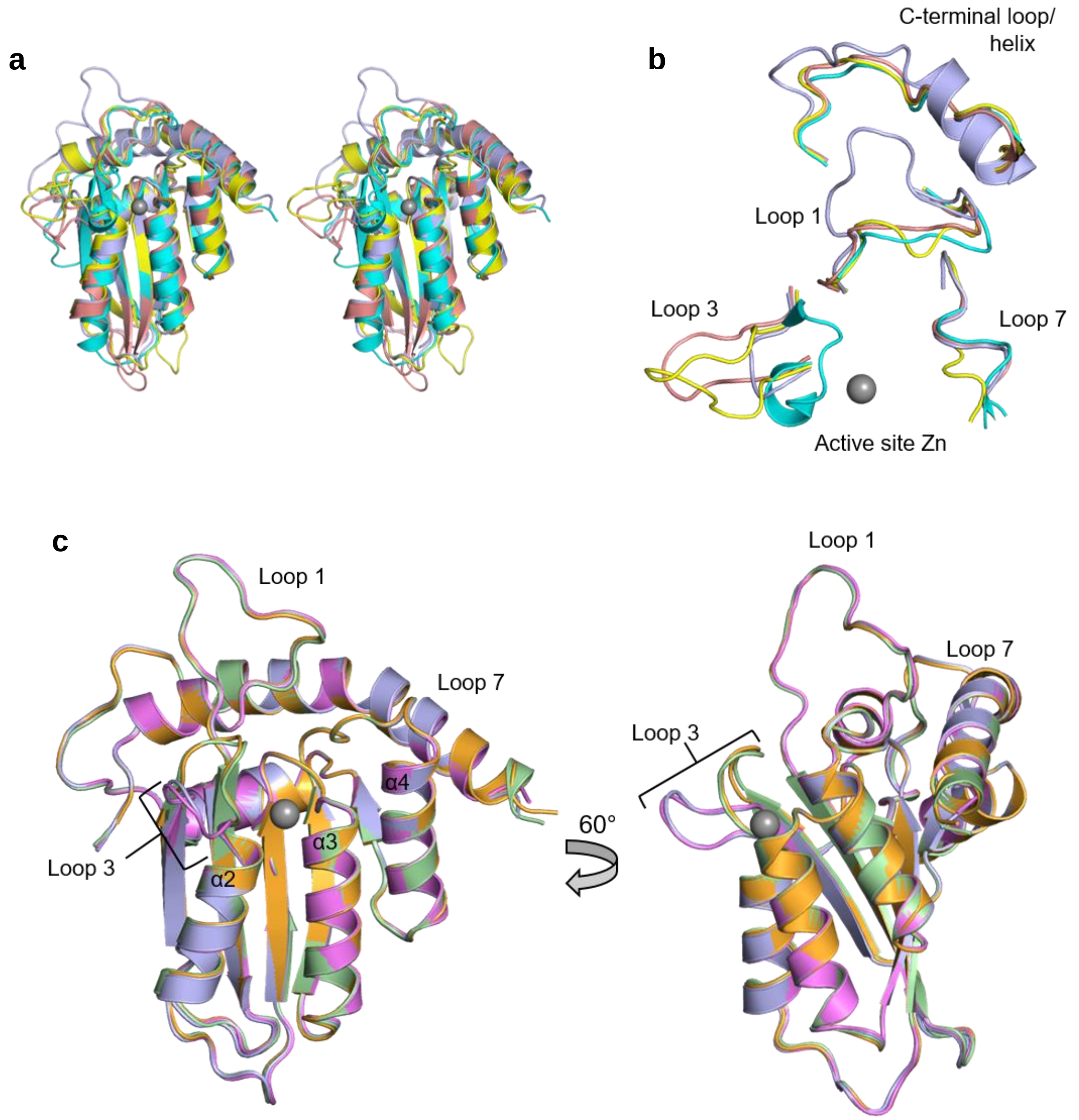

Supplementary Figure 4: Comparison of A3H with other A3 structures.
(a) Stereo view of the structure of A3F and A3G domains superimposed on A3H (light blue). The core APOBEC fold is conserved in A3H and the A3G RNA-binding domain (5K81 [1], RMSD= 0.96 Å, cyan), the A3G catalytic domain (3IQS [2], RMSD= 0.96 Å, yellow), and the A3F catalytic domain (3WUS [3], RMSD= 1.13 Å, salmon). (**b**) Key loop regions from the superposition in **a**. The longer loop 1 in A3H inserts into the narrow major groove of the RNA duplex. (**c**) Superposition of the four independent A3H molecules in the crystal structure. The polypeptides (in contrasting colors) are identical within experimental error excepting loop 3, which adopts two conformations in crystals and does not contact RNA.

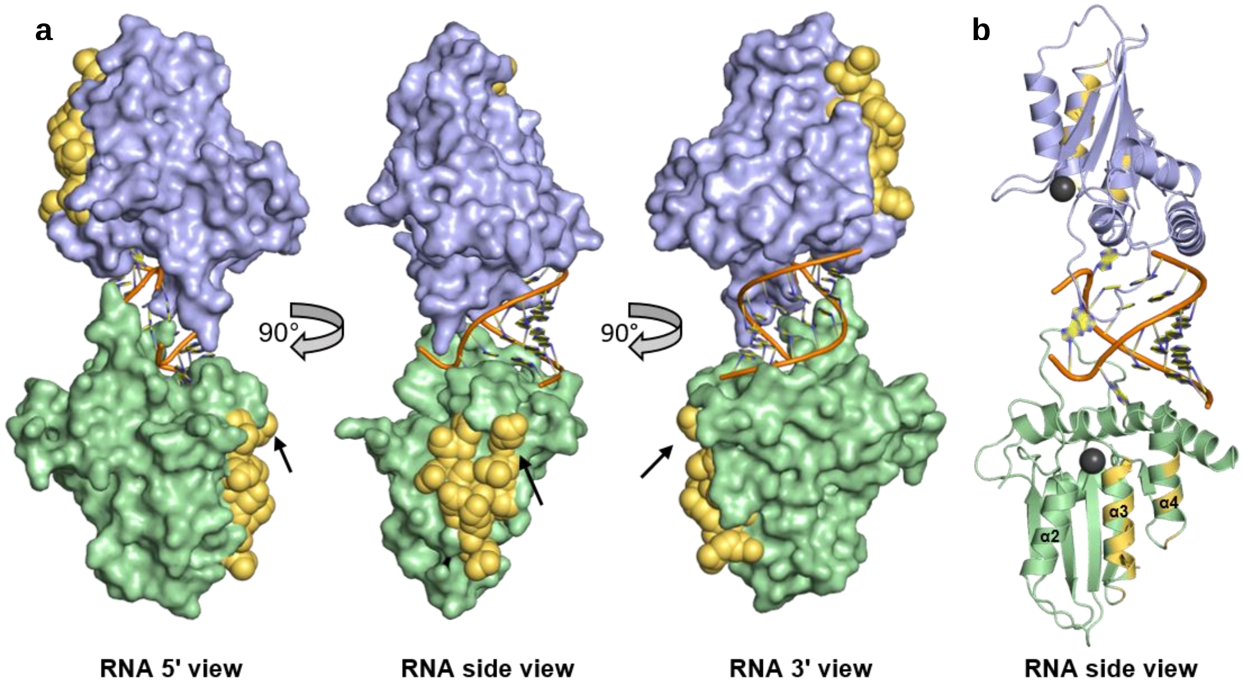

Supplementary Figure 5: Potential A3H-Vif interaction region.
(a) Surface view of the A3H-RNA complex (colored as in Fig 3). Amino acids implicated in Vif binding (86, 87, 90, 91, 93, 94, 96, 97, 100, 121, 125, 129 [4]) are highlighted in yellow on the blue and green A3H molecules with an arrow indicating residue 121 [4,5,6]. **(b)** Cartoon representation of the A3H-RNA complex. Residues implicated in Vif binding (yellow) are located on the outer surfaces of helices α3 and α4.

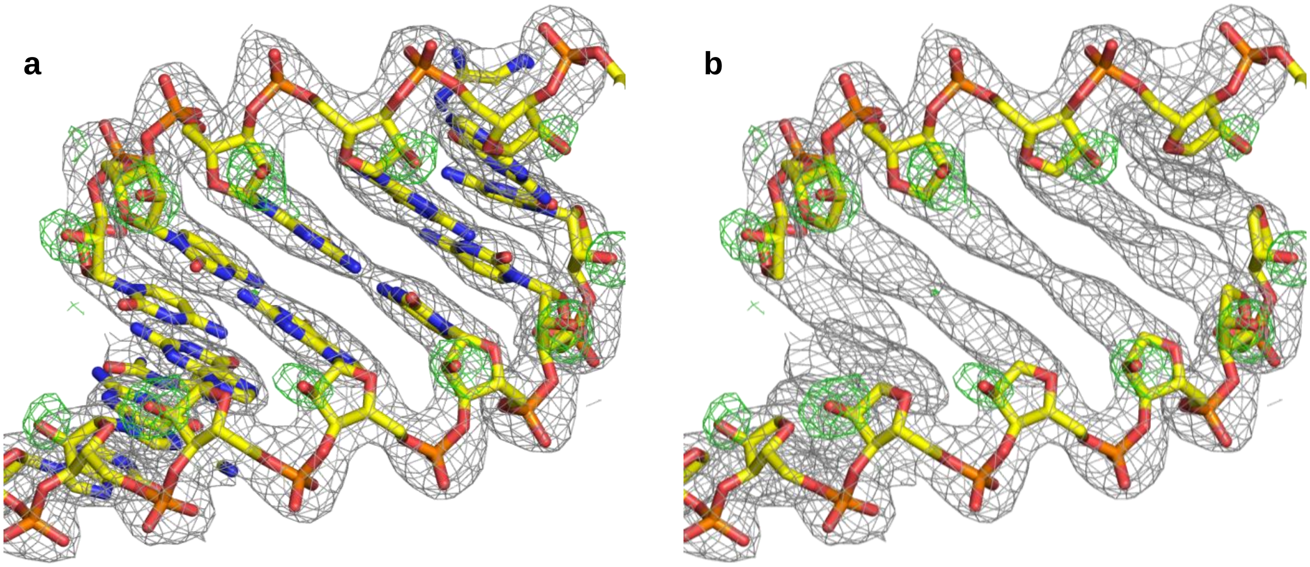

Supplementary Figure 6: Electron density for RNA.
(a) $2F_o$-F_c density (gray contours at 1.5σ) for the RNA duplex with RNA in stick form with yellow C atoms. Density is continuous across each base pair, consistent with a co-purified RNA of heterogeneous sequence. The F_o-F_c "omit" density (green contours at 3σ) is based on a model from which the 2′-OH of each ribose was deleted and demonstrates that both strands of the duplex are RNA and not DNA, consistent with the analysis of extracted nucleic acid (Supplementary Fig. 2d). **(b)** RNA duplex density (as in **a**) with bases removed to illustrate the continuous density across the base pairs.

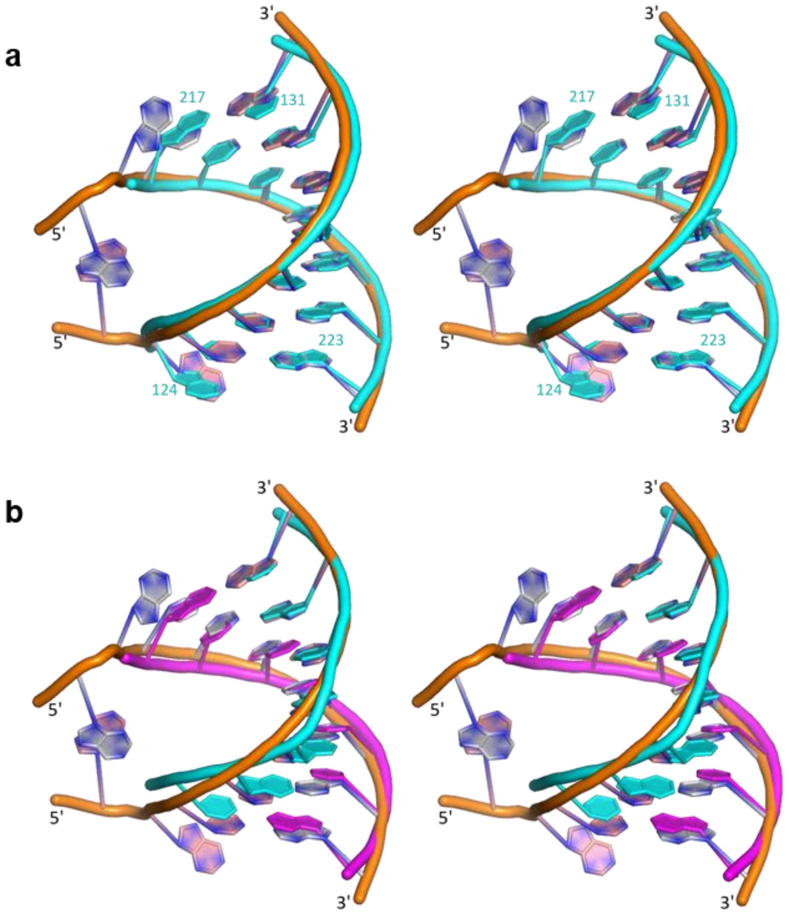

Supplementary Figure 7: Comparison of RNA bound to A3H with structures of nucleic acid duplexes.

(a) Overlay of A3H RNA (orange cartoon) with an RNA stem from the lowest-energy structure in an NMR ensemble of the HIV-1 primer binding site (cyan, PDB 2N1Q [7], RMSD = 0.6 Å for 14 C1' atoms). The strand with uracil bases (nt 124-131) lies within the site of highest frequency binding (Fig. 5a, peak 1) within the HIV-1 genome in virions. **(b)** Overlay with an RNA:DNA heteroduplex 2.3-Å crystal structure (cyan RNA strand, magenta DNA strand, 1FIX [8], RMSD = 0.9 Å). The stereo images illustrate the A3H selectivity for A-form duplex. The sugar puckers are C3′-endo for all RNA strands in the APOBEC3H-RNA complex, in the NMR structure (PDB 2N1Q), and in the RNA strand of the RNA-DNA heteroduplex (PDB 1FIX). In the heteroduplex DNA strand, the sugar pucker is C3′-endo for the seven nucleotides at the 5′ end, and O4′-endo, C2′-endo and C2′-endo, respectively, for the three nucleotides at the 3′ end.

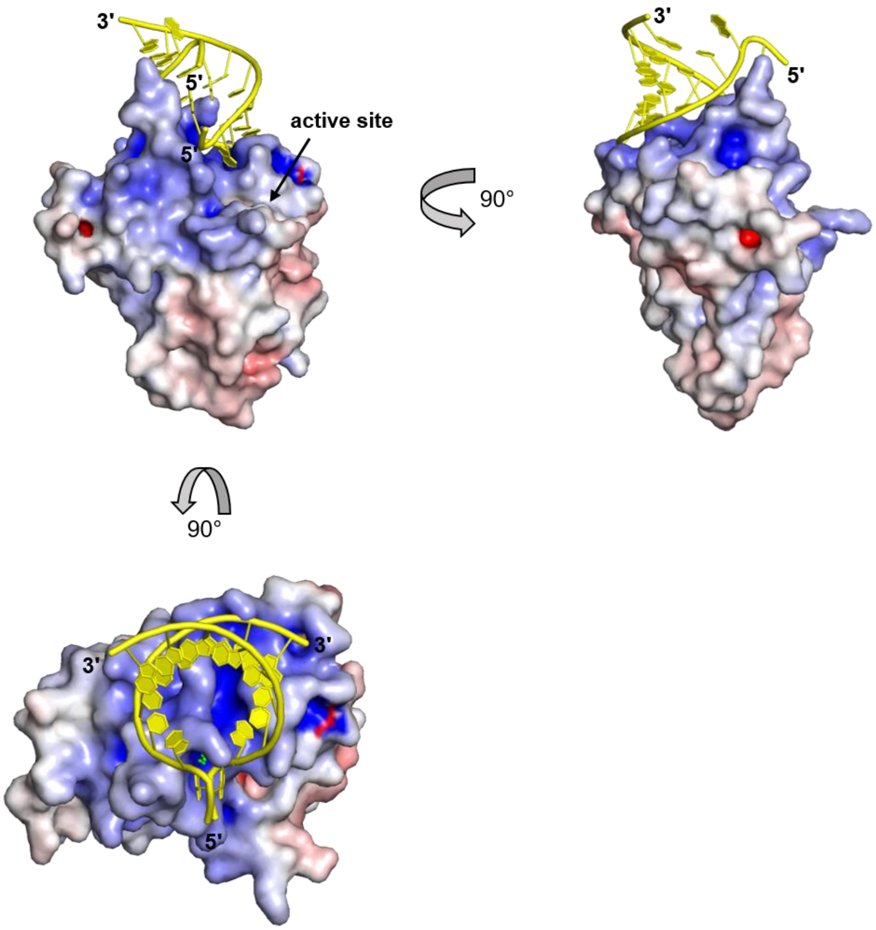

Supplementary Figure 8: A3H electrostatic surface potential.
Perpendicular views of the A3H electrostatic surface potential (colored from red to blue, -10 to +10 kT/e) with RNA (yellow cartoon) bound to the most highly basic surface.

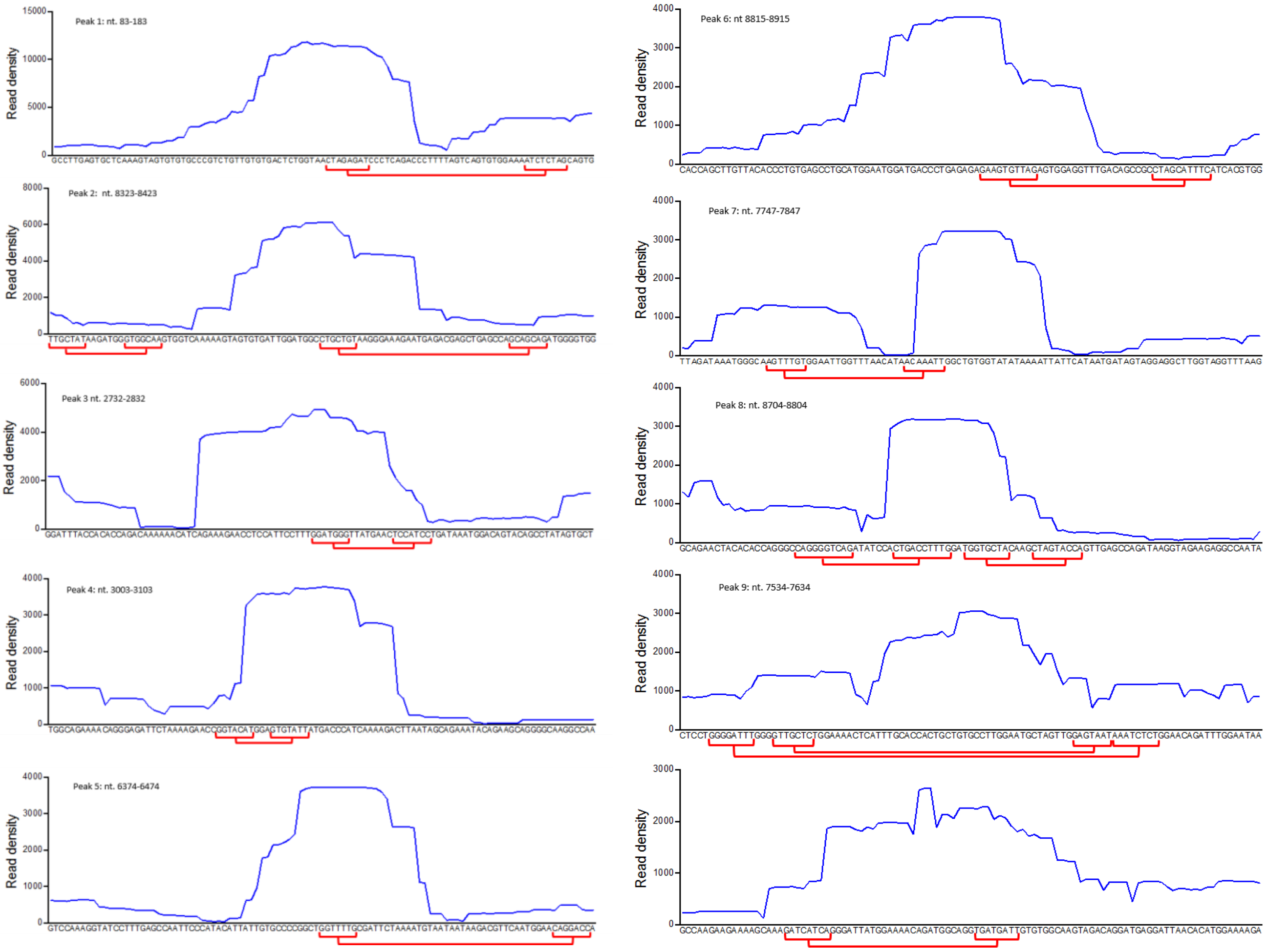

Supplementary Figure 9: Nucleotide sequences of the peak sites of highest read densities in the CLIP-Seq analysis of huA3H binding to HIV-1 RNA in virions.

Each plot corresponds to a high-frequency peak (Fig. 5a); red brackets indicate complementary regions capable of forming a stem with ≥7 base pairs.

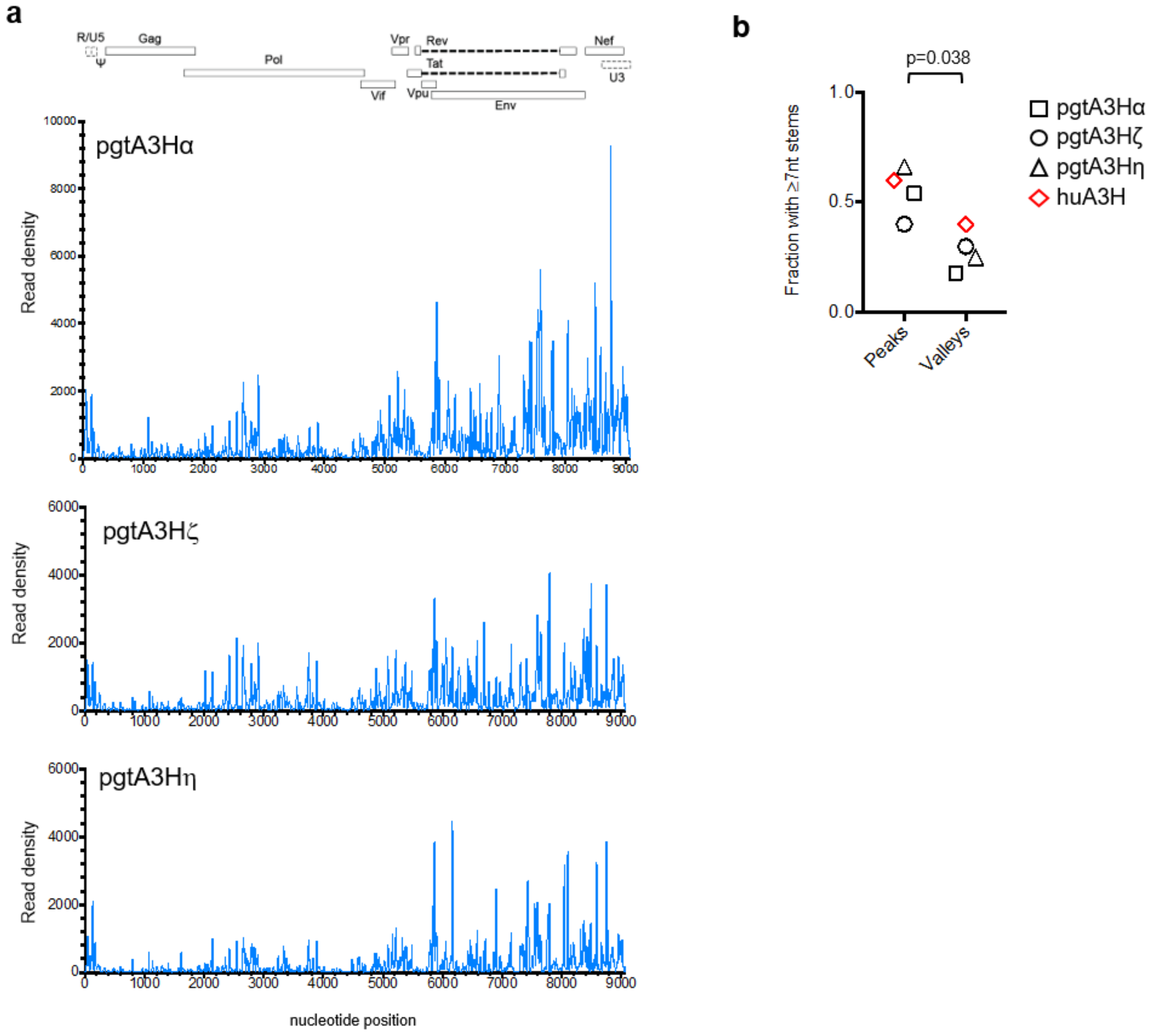

Supplementary Figure 10: Analysis of pgtA3H variant binding to HIV-1 RNA in infected cells.

(a) CLIP-Seq analysis of A3-RNA cross-linked complexes that were immunoprecipitated from HIV-$1_{NL4.3\Delta Vif}$ infected cells stably expressing 3xHA-tagged pgtA3H variants that had been fed with 4SU and UV-irradiated. Frequency distribution of nucleotide occurrence (read density) in reads mapped to the HIV-$1_{NL4.3}$ genome. A schematic diagram of the HIV-1 genome is shown above. **(b)** Frequency with which ≥7-nt duplexes are predicted to occur within 101-nt RNA elements containing the 10 sites with high-frequency binding, and 101-nt elements with low-frequency binding for each pgtA3H variant (from **a**) and huA3H[9].

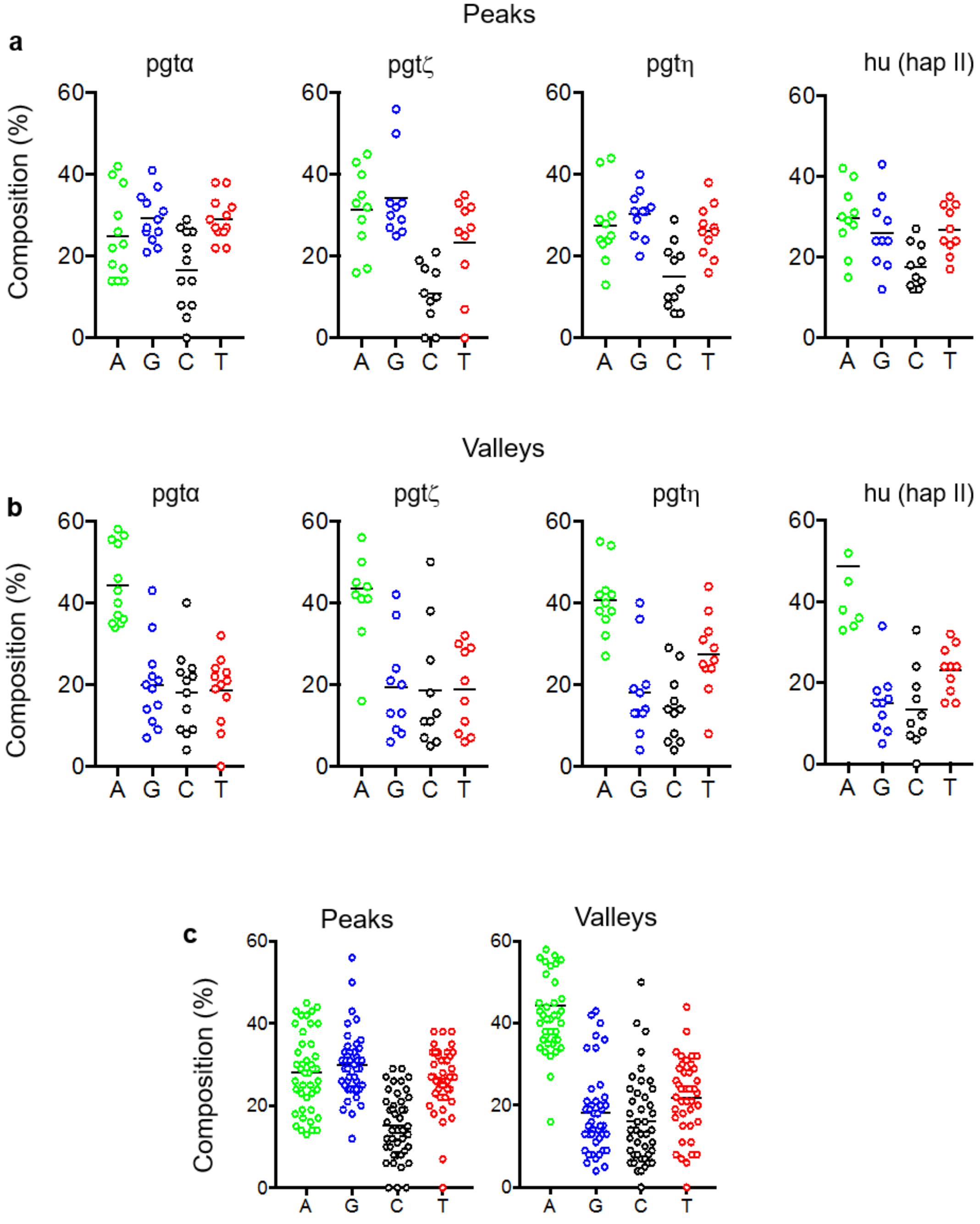

Supplementary Figure 11: Nucleotide composition of A3H peaks and valleys in infected cells. (a) The nucleotides with the highest read density within each of the 10 peaks identified for each pgtA3H variant and huA3H (Supplementary Figure 10) were selected and the base composition determined. **(b)** For comparison, we identified short valleys as stretches of nucleotides with identical length to the peak tips in a proximal region of the HIV-1 genome. In each CLIP-seq experiment, approximately 2-3 peaks had wide tips that rendered the identification of short valleys in the vicinity of those peaks impossible and therefore distal regions of the genome were used. **(c)** Nucleotide composition of peak tips and short valleys compiled from all A3H proteins analyzed in **a** and **b**.

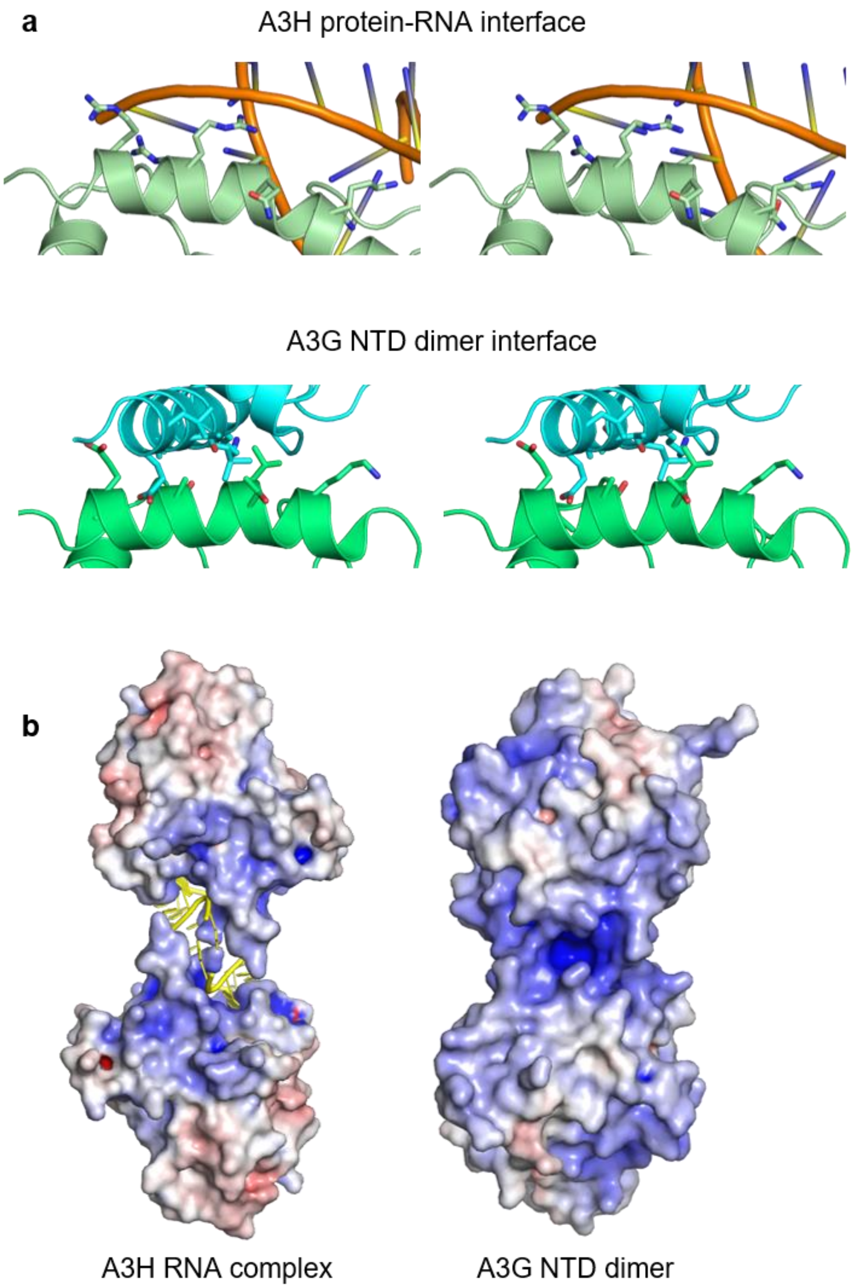

Supplementary Figure 12. Comparison of A3H and A3G.
(a) C-terminal helices of A3H (top) and the A3G RNA-binding NTD[1] dimer (bottom) shown in stereo, based on superposition of A3H and the green subunit of the A3G dimer. Arginine and lysine side chains on the A3H C-terminal helix interact directly with the phosphate backbone of the RNA duplex whereas the hydrophobic surface of the C-terminal helix of the A3G NTD[1] (bottom) is buried in the dimer interface with the cyan subunit. **(b)** Basic surfaces of A3H and A3G. RNA binds to the basic surfaces of two A3H molecules (left), which do not contact one another. In contrast, the most electropositive surface of the A3G NTD[1] (right) is created in the dimer, which is formed through contact of hydrophobic surfaces as shown in **a**. The electrostatic surface potentials are colored from red to blue (-10 to +10 kT/e).

Supplementary Table 1. Codon optimized DNAs and primers

**Codon optimized DNAs**	
**huA3H (hap II)**	ATGGCTGCTCTTTTAACAGCCGAAACATTCCGCTTACAGTTTAACAACAAGCGCCGCCTCAGAAGGCCTTACTAC<br>CCGAGGAAGGCCCTCTTGTGTTACCAGCTGACGCCGCAGAATGGCTCCACGCCCACCAGAGGCTACTTTGAAAAC<br>AAGAAAAAGTGCCATGCAGAAATTTGCTTTATTAACGAGATCAAGTCCATGGGACTGGACGAAACGCAGTGCTA<br>CCAAGTCACCTGTTACCTCACGTGGAGCCCCTGCTCCTCCTGTGCCTGGGAGCTGGTTGACTTCATCAAGGCTCAC<br>GACCATCTGAACCTGCGCATCTTCGCCTCCCGCCTGTACTACCACTGGTGCAAGCCCCAGCAGGACGGGCTGCGG<br>CTTCTGTGTGGATCCCAGGTCCCGGTGGAGGTCATGGGCTTCCCAGAGTTTGCTGACTGCTGGGAAAACTTTGTGG<br>ACCACGAGAAACCGCTTTCCTTCAACCCCTATAAGATGTTAGAGGAGCTAGATAAAAACAGTCGAGCCATAAAG<br>CGACGGCTTGACAGGATAAAGCAGTCCTAAC
**pgtA3Hα**	ATGGCTCTGCTAACAGCCAAAACATTCAGCTTACAGTTTAACAACAAGCGCCGAGTCAACAAGCCTTACTACCCG<br>AGGAAGGCCCTCTTGTGTTACCAGCTGACGCCGCAGAATGGCTCCACGCCTACCAGAGGCCACTTAAAAAACAA<br>GAAAGAGGACCATGCAGAAATTCGCTTTATTAACAAGATCAAGTCCATGGGACTGGACGAAACCCAGTGCTACC<br>AAGTCACCTGTTACCTCACGTGGAGCCCCTGTCCCTCCTGTGCCGGGGAGCTGGTTGACTTCATCAAGGCTCACCG<br>CCATCTGAACCTGCGCATCTTCGCCTCCCGCCTGTACTACCACTGGCGCCCGAACTATCAGGAGGGGCTGCTGCTT<br>CTGTGTGGATCCCAGGTCCCAGTGGAGGTCATGGGCCTCCCAGAGTTTACTGACTGCTGGGAAAACTTTGTGGAC<br>CACAAGGAACCGCCTTCCTTCAACCCCTCTGAGAAGTTAAAGGAGCTAGATAAAAACAGCCAAGCCATAAAGCG<br>ACGGCTTGAAAGGATAAAGTCCCGGAGTGTGGATGTTTTAGAGAATGGCTTAAGAAGTTTGCAGCTTGGACCCGT<br>AACCCCCTCATCGTCAATACGCAACTCAAGATGA
**pgtA3Hη**	ATGGCTCTGCTAACAGCCAAAACATTCAGCTTACAGTTTAACAACAAGCCCCGTGTCAACAAGCCTTACTACCCG<br>AGGAAGGCCCTCTTGTGTTACCAGCTGACGCCGCAGAATGGCTCCACGCCTACCAGAGGCCACTTAAAAAACAA<br>GAAAGAGGACCATGCAGAAATTCGCTTTATTAACGAGATCAAGTCCATGGGACTGGACGAAACCCAGTGCTACC<br>AAGTCACCTGTTACCTCACGTGGAGCCCCTGTCCCTCCTGTGCCGGGGAGCTGGTTGACTTCATCAAGGCTCACCG<br>CCATCTGAACCTGCGCATCTTCGCCTCCCGCCTGTACTACCACTGGCGCCCGAACTATCAGGAGGGGCTGCTGCTT<br>CTGTGTGGATCCCAGGTCCCAGTGGAGGTCATGGGCCTCCCAGAGTTTACTGACTGCTGGGAAAACTTTGTGGAC<br>CACAAGGAACCGCCTTCCTTCAACCCCTCTGAGAAGTTAAAGGAGCTAGATAAAAACAGCCAAGCCATAAAGCG<br>ACGGCTTGAAAGGATAAAGTCCCGGAGTGTGGATGTTTTAGAGAATGGCTTAAGAAGTTTGCAGCTTGGACCCGT<br>AACCCCCTCATCGTCAATACGCAACTCAAGAGGCGGCCGCTGA
**pgtA3Hζ**	ATGGCTCTGCTAACAGCCAAAACATTCAGCTTACAGTTTAACAACAAGCGCCGTGTCAACAAGCCTTACTACCCG<br>AGGAAGGCCCTCTTGTGTTACCAGCTGACGCCGCAGAATGGCTCCACGCCTACCAGAGGCCACTTAATAAACAAG<br>AAAGAGGACCATGCAGAAATTCGCTTTATTAACGAGATCAAGTCCATGGGACTGGACGAAACCCAGTGCTACCA<br>AGTCACCTGTTACCTCACGTGGAGCCCCTGTCCCTCCTGTGCCGGGGAGCTGGTTGACTTCATCAAGGCTCACCGC<br>CATCTGAACCTGCGCATCTTCGCCTCCCGCCTGTACTACCACTGGCACCCGAACTATCAGGAGGGGCTGCTGCTTC<br>TGTGTGGATCCCAGGTCCCAGTGGAGGTCATGGGCCTCCCAGAGTTTACTGACTGCTGGGAAAACTTTGTGGACC<br>ACAAGGAACCGCCTTCCTTCAACCCCTCTGAGAAGTTAAAGGAGCTAGATAAAAACAGCCAAGCCATAAAGCGA<br>CGGCTTGAAAGGATAAAGTCCCGGAGTGTGGATGTTTTAGAGAATGGCTTAAGAAGTTTGCAGCTTGGACCCGTA<br>ACCCCCTCATCGTCAATACGCAACTCAAGAGGCGGCCGCTGA
**UDG**	ATGGCAAATGAGTTGACGTGGCACGATGTATTGGCGGAAGAGAAGCAACAACCCTATTTCTTGAATACGCTTCAA<br>ACGGTCGCTTCGGAGCGCCAGAGCGGCGTGACGATCTACCCACCGCAAAAAGACGTGTTTAACGCCTTTCGCTTC<br>ACTGAACTTGGGGACGTTAAAGTCGTGATTCTTGGGCAAGACCCCTATCATGGGCCAGGACAAGCGCATGGCTTG<br>GCGTTTAGCGTCCGCCCGGGTATCGCCATTCCACCGTCGTTACTTAATATGTACAAGGAACTGGAGAATACGATC<br>CCTGGTTTCACTCGCCCAAACCACGGCTATCTGGAAAGCTGGGCTCGTCAGGGCGTCCTGCTGCTTAACACAGTA<br>TTGACGGTTCGTGCAGGTCAGGCTCACAGTCACGCATCCTTAGGTTGGGAAACCTTTACTGACAAAGTCATCAGT<br>CTTATCAACCAGCACCGTGAGGGGGTAGTTTTTTTGCTTTGGGGCTCTCATGCCCAAAAAAAGGGTGCTATTATCG<br>ACAAGCAGCGCCATCACGTCCTTAAAGCACCACATCCATCCCCGTTGTCCGCGCATCGCGGGTTTTTTGGGTGTAA<br>CCACTTTGTTCTTGCCAATCAATGGCTTGAGCAACGTGGAGAGACTCCCATCGACTGGATGCCCGTTTTACCCGCT<br>GAATCTGAA
**Primers used for synthesis of pigtailed macaque cDNA**	
**macA3H-F**	5′-TATTGCAAGCTTGCCATGGCTCTGCTAACAGCCAAAAC-3′
**macA3H-R**	5′-GTCTTAGTTAACGCGGCCGCCTCTTGAGTTGCGTATTGACGATGAGG-3′
**Primers for pgtA3Hα E56A mutagenesis (mutated codon underlined)**	
**α E56A-F**	5′-AACAAGAAAGAGGACCATGCAGCAATTCGCTTTATTAACAAGATC-3′
**α E56A-R**	5′-GATCTTGTTAATAAAGCGAATTGCTGCATGGTCCTCTTTCTTGTT-3′

Supplementary Note 1. Uncropped gel images for Supplementary Fig. 3

Supplementary Fig. 3a uncropped gels for huA3H (hap II) and pgtA3Hα samples (top) and pgtA3Hη and pgtA3Hζ (bottom).

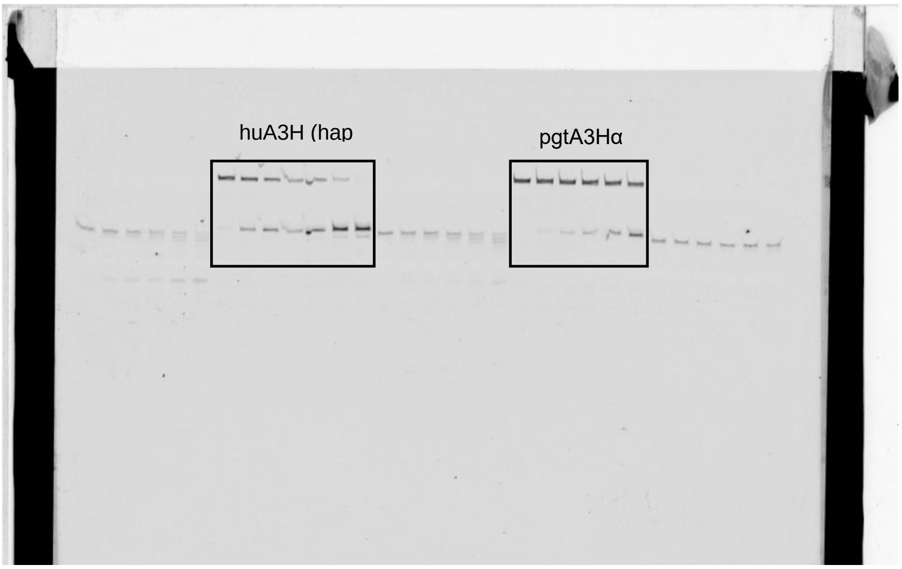

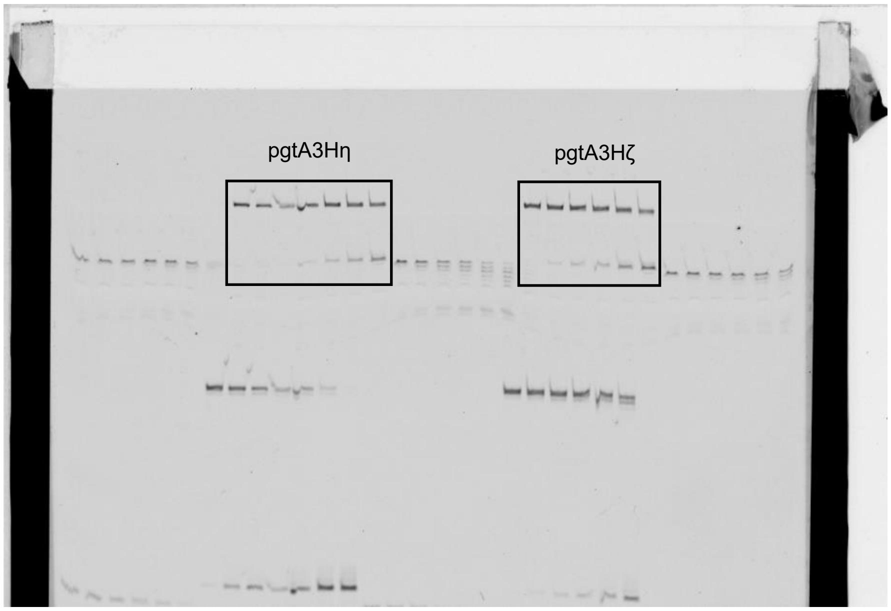

Supplementary Fig. 3c uncropped gel for all +/- RNase A treated samples for huA3H (left) and pgtA3Hζ (right).

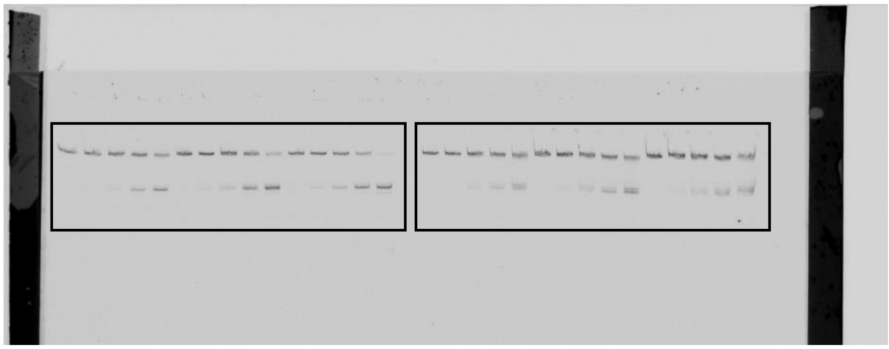

Supplementary References

1 Xiao, X., Li, S.-X., Yang, H. & Chen, X. S. Crystal structures of APOBEC3G N-domain alone and its complex with DNA. *Nat Commun* **7**, 12193, doi:10.1038/ncomms12193 (2016).

2 Holden, L. G. *et al.* Crystal structure of the anti-viral APOBEC3G catalytic domain and functional implications. *Nature* **456**, 121-124, doi:10.1038/nature07357 (2008).

3 Nakashima, M. *et al.* Structural insights into HIV-1 Vif-APOBEC3F interaction. *J Virol* **90**, 1034-1047, doi:10.1128/JVI.02369-15 (2016).

4 Zhen, A., Wang, T., Zhao, K., Xiong, Y. & Yu, X. F. A single amino acid difference in human APOBEC3H variants determines HIV-1 Vif sensitivity. *J Virol* **84**, 1902-1911, doi:10.1128/JVI.01509-09 (2010).

5 Ooms, M., Letko, M. & Simon, V. The structural interface between HIV-1 Vif and human APOBEC3H. *J Virol* **91**, doi:10.1128/JVI.02289-16 (2017).

6 Nakashima, M. *et al.* Mapping Region of Human Restriction Factor APOBEC3H Critical for Interaction with HIV-1 Vif. *J Mol Biol* **429**, 1262-1276, doi:10.1016/j.jmb.2017.03.019 (2017).

7 Keane, S. C. *et al.* RNA structure. Structure of the HIV-1 RNA packaging signal. *Science* **348**, 917-921, doi:10.1126/science.aaa9266 (2015).

8 Horton, N. C. & Finzel, B. C. The structure of an RNA/DNA hybrid: a substrate of the ribonuclease activity of HIV-1 reverse transcriptase. *J Mol Biol* **264**, 521-533, doi:10.1006/jmbi.1996.0658 (1996).

9 York, A., Kutluay, S. B., Errando, M. & Bieniasz, P. D. The RNA binding specificity of human APOBEC3 proteins resembles that of HIV-1 nucleocapsid. *PLoS Pathog* **12**, e1005833, doi:10.1371/journal.ppat.1005833 (2016).
